# Endogenous inhibitors of PP2A activate oncogenic and DNA damage response kinases in glioblastoma

**DOI:** 10.1016/j.canlet.2026.218325

**Published:** 2026-02-20

**Authors:** John Ryan Jacob, Shahid M. Nimjee, J. Bradley Elder, Arnab Chakravarti, Kamalakannan Palanichamy

**Affiliations:** a Department of Radiation Oncology, The Ohio State University College of Medicine and Comprehensive Cancer Center, Columbus, OH, 43210, USA; b Department of Neurosurgery, The Ohio State University Wexner Medical Center, Columbus, OH, 43210, USA

**Keywords:** GBM, PP2A, SET, ANP32A, CIP2A, Radiation

## Abstract

Glioblastomas (GBMs) exhibit constitutive activation of oncogenic kinase signaling pathways, contributing to tumor aggressiveness and resistance to therapy. Kinase inhibitors have demonstrated limited efficacy against GBMs, primarily due to the tumors’ ability to adapt to diverse stimuli and effectively rewiring critical downstream signaling networks. This remarkable adaptability underscores the pressing need for novel therapeutic strategies that sustain the inhibition of oncogenic kinase signaling in GBM. This study sought to elucidate the mechanisms by which GBMs maintain the constitutive activation of oncogenic kinase signaling under the surveillance of intact tumor suppressor protein phosphatase 2A (PP2A). We identify that GBMs inhibit PP2A activity through overexpression of endogenous inhibitors (EIPs), including ANP32A, CIP2A, and SET. Inhibition of these EIPs restores PP2A activity, disrupting oncogenic kinase activation and transcription factor signaling, reducing tumor formation. Furthermore, CRISPR-Cas9 EIP silencing enhances PP2A’s ability to target DNA damage response kinases ATR and ATM, sensitizing tumors to radiation by impairing DNA repair and cell cycle checkpoint control. These findings reveal the therapeutic potential of activating PP2A in GBMs, both as a standalone strategy and in combination with radiation.

## 1. Introduction

Glioblastoma (GBM), the most common primary brain cancer in adults, is a lethal malignancy due to its aggressive nature. GBMs persistently activate diverse serine/threonine signaling networks, facilitating proliferation, metabolic reprogramming, and treatment resistance [1]. Genetic alterations within the receptor tyrosine kinase (RTK)/Ras/PI3K pathway and the upregulation of heterogenous and intact RTKs maintain these pathways [2-7]. This redundancy in signaling enhances signaling plasticity and adaptability, making anti-RTK therapies ineffective [5,8]. Targeting oncogenic signaling in GBM requires an innovative approach to short-circuit these multiple signaling inputs. Evidence exists that GBMs circumvent the serine/threonine phosphatase, protein phosphatase 2A (PP2A), a tumor suppressor that counteracts constitutively activated serine/threonine signaling networks in GBMs [9]. PP2A comprises over 68 heterotrimer complexes with three subunits: structural (PP2A-A), catalytic (PP2A-C), and regulatory (PP2A-B) [10]. While PP2A is genetically suppressed in endometrial, colon, and lung cancers, it remains intact in GBMs [11,12]. We aimed to elucidate the mechanisms GBMs use to evade PP2A surveillance of oncogenic serine/threonine signaling.

## 2. Materials and methods

### 2.1. Experimental model and subject details

We conducted this study in accordance with The Ohio State University Institutional Review Boards for IRB (2009C0065 and 2014C0115), IACUC (2009A0127), and IBC (2009R0169).

### 2.2. Methods details

#### 2.2.1. Cell lines and tissue culture

U87-MG (HTB-14), LN18 (CRL-2610), LN229 (CRL-2611), and T98G (CRL-1690) were purchased from American Type Culture Collective (ATCC, Manassas, VA, USA). OSU2, MGH8, GBM, ACPK1, ACPK4, and ACPK8 were propagated from GBM tissue authenticated by neuropathologists. All cells were cultured in DMEM, high glucose, pyruvate (Thermo: 11995073) supplemented with 10% FBS (Invitrogen) and 1% antibiotic–antimycotic (Thermo: 15240062). All cells were cultured at 37 °C under a gas phase of 95% air and 5% CO2 and were tested for mycoplasma contamination (ATCC: 30-1012K) periodically throughout the study period. All studies were conducted within 10 cell line passages and were authenticated using STR profiles.

#### 2.2.2. In vivo studies

About 1x10^5^ U87-MG isogenic cells resuspended in serum free DMEM medium (3 mL) were intracranially injected into the brain of athymic nu/nu female mice (6–8 weeks old). Mice were monitored for weight loss or neuro-symptoms until moribund, at which point they were sacrificed. Brains were isolated and fixed in 4% formaldehyde, embedded in paraffin, and sectioned for H&E. Targeted radiation was delivered using the Small Animal Radiation Research Platform (SARRP; Xstrahl) ten days post implantation. 3 doses of 2 Gy radiation were administered every other day for one week. Radiation was delivered across a 5 mm field centered on the burr hole.

#### 2.2.3. Statistical analysis

All results in this study are presented as mean ± SD of at least three independent experiments. The sample size of each experiment is indicated in the corresponding Figure legend. One-way ANOVA was used when several groups were being compared or unpaired Student’s *t*-test (two-tailed) when two groups were being compared. Log-rank test was used to compare survival curves. P values less than 0.05 were considered statistically significant.

#### 2.2.4. Supplementary methods

Additional details on methods including Western blotting, CRISPR-cas9 gene editing, RT-qPCR, NFκB ELISA, Clonogenics, COMET assay, Annexin V/PI, RNA-Seq analysis, Proximity Biotin Ligase, Cell cycle synchronization and analysis, Immunofluorescence, Proximity ligation assay, β-galactosidase assay, and Immunohistochemistry is included in the supplemental information.

## 3. Results

### 3.1. Aberrant expression of PP2A inhibitory proteins promotes GBM tumorigenesis

**Fig. 1.**
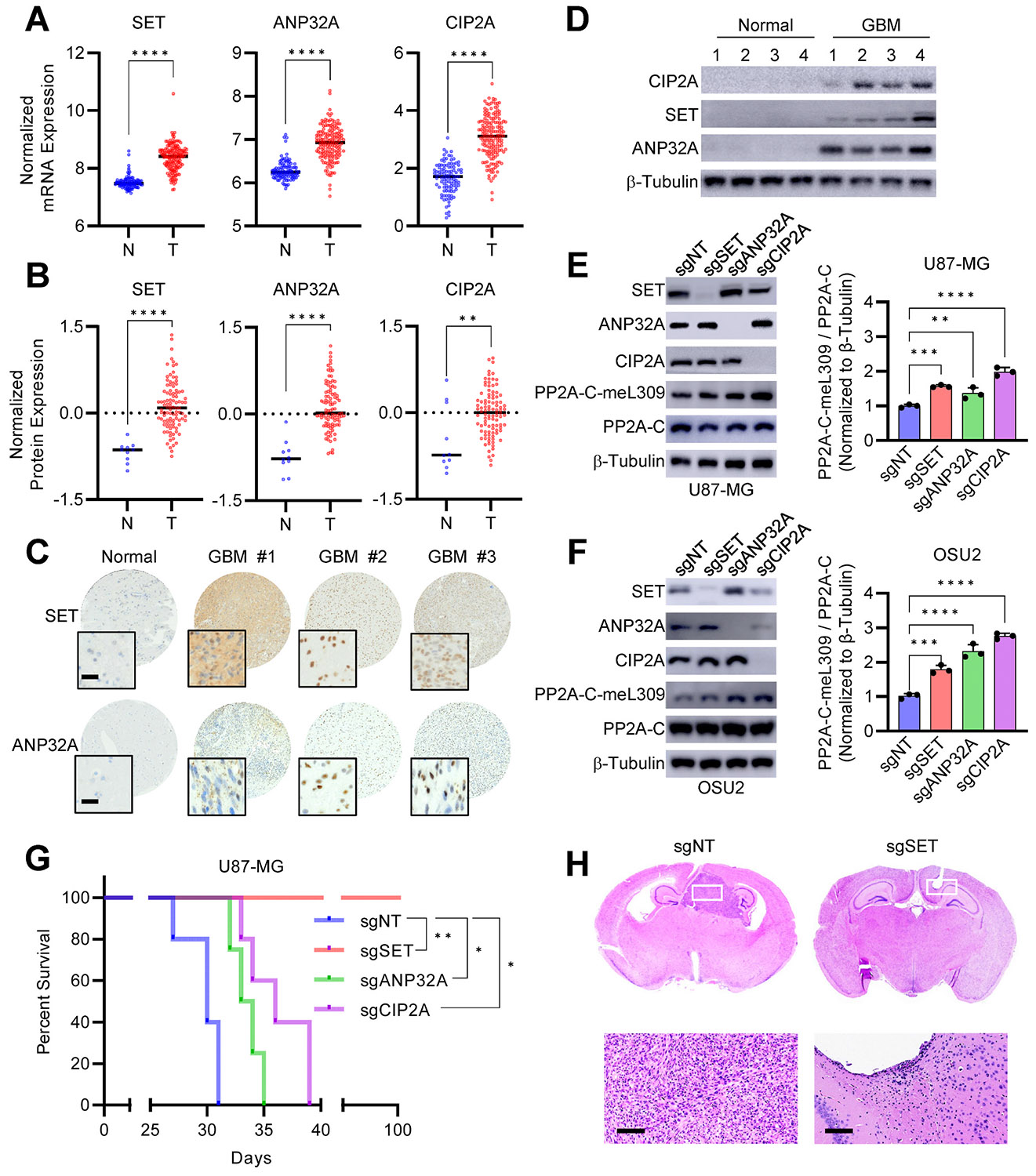
Aberrant expression of PP2A inhibitory proteins promotes GBM tumorigenesis. (a) Normalized mRNA expression of indicated genes in normal brain (GTEx, n = 105) and GBM (TCGA, n = 172) tissues following RNA-seq normalization as described in Supplemental Methods. (b) Normalized protein expression values of indicated proteins in normal brain (n = 10) and GBM (n = 99) tissues, as reported in Wang et al., Cancer Cell (2021). (c) Immunohistochemical staining of indicated proteins in normal brain and GBM biopsies. Scale bars: 50 μm. (d) Assessment of indicated protein levels using western blotting of normal brain tissues and GBM specimens. (e) Left: Assessment of indicated protein levels using western blotting of U87-MG sgNT, sgSET, sgANP32A, and sgCIP2A cell lines. Right: Quantification of PP2A-C methylation levels normalized to total PP2A-C and β-Tubulin (n = 3). (f) Left: Assessment of indicated protein levels using western blotting of OSU2 sgNT, sgSET, sgANP32A, and sgCIP2A cell lines. Right: Quantification of PP2A-C methylation levels normalized to total PP2A-C and β-Tubulin (n = 3). (g) Kaplan-Meier curve of mouse survival following intracranial implantation of U87-MG sgNT, sgSET, sgANP32A, and sgCIP2A cells (n = 5). (h) Top: Representative H&E images of tumors formed by U87-MG sgNT and sgSET cell lines. Bottom: Zoomed in images of H&E scans. Scale bars: 200 μm. Data represents mean ± SD. *p < 0.05, **p < 0.01, ***p < 0.001. See also Fig. S1.

We hypothesized that GBMs sustain aberrant serine/threonine signaling, in part through post-translational suppression of protein phosphatase 2A (PP2A). To identify potential mechanisms of inhibition, we profiled transcriptional and translational regulation of PP2A subunits and regulators in GBM versus normal brain tissue (Figure. S1, A and B). Three endogenous PP2A inhibitors—SET, ANP32A, and CIP2A—were overexpressed in GBM specimens (Fig. 1A and B). These inhibitors suppress PP2A activity by sequestering subunits or complexes, providing a plausible mechanism for phosphatase suppression [13,14]. Immunohistochemistry confirmed elevated SET and ANP32A in GBM tissues (Fig. 1C), though CIP2A staining was unreliable. Western blotting corroborated increased EIP protein levels in GBM tissues and cell lines (Fig. 1D; Fig. S1C), indicating that elevated EIPs are a consistent GBM feature. Further, protein expression of SET, ANP32A, and CIP2A exhibits strong to moderate positive correlation across GBM specimens (Figure S1, K-M), indicating that these endogenous PP2A inhibitors are frequently co-expressed rather than independently regulated. This co-ordinated elevation suggests that PP2A suppression in GBM may be achieved through the concurrent action of multiple EIPs.

In contrast, GBMs and normal brain tissues possess decreased expression of enzymes modulating PP2A via alternative post-translational modifications, including methylation (LCMT-1, PME-1), [15], proline isomerization (PIN1) [16], and cell cycle-related inhibitors (ENSA, ARPP19) [17] (Figure S1, D-I). These findings point to EIP overexpression as the predominant mechanism of PP2A suppression in GBM.

To evaluate the potential oncogenic function of the EIPs, we employed CRISPR-Cas9-mediated gene editing to ablate ANP32A, CIP2A, and SET in the established U87-MG and the patient-derived OSU2 cell lines (Fig. 1E and F). As PP2A-C subunit methylation is essential for the assembly and activity of a subset of PP2A holoenzymes [18], we used it as a surrogate marker of phosphatase activation. Given the observed instability of PP2A-C methylation post-lysis (Fig. S1J), we implemented immediate quenching protocols to preserve physiological methylation states [19]. Deletion of EIPs resulted in enhanced PP2A-C methylation in both models, consistent with EIP-mediated suppression of PP2A activity in GBM (Fig. 1E and F).

To assess the functional consequences of EIP loss *in vivo*, we performed orthotopic implantation of EIP knockout cells into immunocompromised mice. ANP32A and CIP2A loss modestly extended median survival (13% and 20%, respectively), whereas SET loss completely abrogated tumor formation, with no morbidity or histological evidence of neoplasia at 100 days (Fig. 1G and H). These data suggest that SET, ANP32A, and CIP2A may cooperatively suppress PP2A and promote tumor progression.

### 3.2. Proteomic analysis of the PP2A interactome identifies oncogenic signaling hubs regulated by EIPs

We next assessed the phospho-protein networks protected by EIP-dependent PP2A inhibition. To measure the PP2A interactome, we fused the second-generation biotin ligase (BioID2) to the N-terminal of the PP2A-Aα subunit (BioID2-PP2A-A) enabling *in situ* labeling of proximal interactors without the disruption associated with cell lysis [20,21]. The fusion protein displayed a subcellular localization similar to endogenous PP2A-Aα and retained the ability to associate with the PP2A catalytic (C) and regulatory (B) subunits, as assessed by streptavidin pulldown, immunoprecipitation, and proximity ligation assays (Figure S2, A-E). These data indicate that the BioID2–PP2A-Aα fusion is competent for incorporation into PP2A complexes and suitable for proximity labeling of PP2A-associated proteins.

**Fig. 2.**
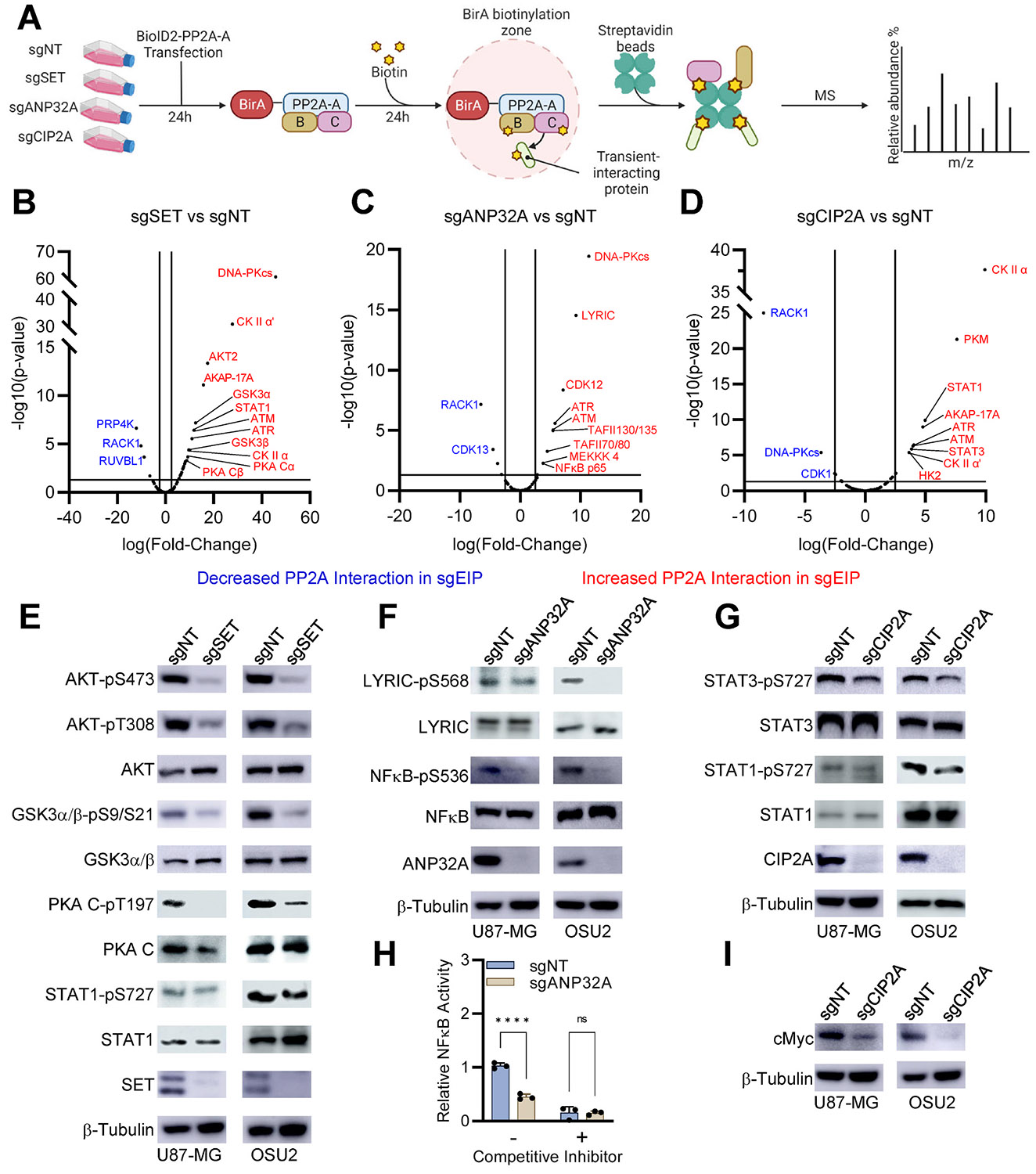
Proteomic analysis of the PP2A interactome identifies oncogenic signaling nodes controlled by EIPs. (a) Experimental workflow of the proximity biotin ligase BioID2-PP2A-Aα fusion protein utilized to map the PP2A interactome in U87-MG sgNT, sgSET, sgANP32A, and sgCIP2A cell lines. (b–d) Volcano plots of proximity labeling proteomics data showing differential PP2A interactions with kinases and transcription factors in U87-MG sgNT cells versus (b) sgSET cells, (c) sgANP32A cells, and (d) sgCIP2A cells (n = 3 biological replicates per condition). The x-axis indicates log2 fold change in total spectral count–normalized protein abundance, and the y-axis indicates −log10 p-value from a two-sided Student’s t-test. Dashed lines denote thresholds of ∣log2 fold change∣ ≥ 1 and p < 0.05. (e-g) Assessment of indicated protein levels using Western blot of sgNT cells and (e) sgSET cells; (f) sgANP32A cells; and (g) sgCIP2A cells. (h) NFκB p65 promoter binding ELISA in U87-MG sgNT and sgANP32A cell lines (n = 3). (i) Assessment of cMyc levels in sgNT and sgCIP2A cell lines. Data represents mean ± SD. *p < 0.05, **p < 0.01, ***p < 0.001 versus sgNT cell lines. See also Figs. S2-4 and Table S1.

We proceeded to map the PP2A interactome by performing unbiased mass spectrometry on BioID2-PP2A-Aα-expressing control and EIP knockout cells (Fig. 2A; Table S1). In knockouts, 367–435 proteins showed ≥2-fold increased PP2A interaction, while 393–471 proteins decreased in association (Figure S2, G and H). To understand the role of EIP-dependent PP2A inhibition on oncogene-induced signaling, we filtered the proteomics data to include kinases and transcription factors (Fig. 2B-D; Figure S2, I and J). We subsequently investigated whether the increased interaction between PP2A and oncogenic kinases and transcription factors alters functionally relevant phosphorylation levels.

SET loss enhanced PP2A binding to Akt, GSK3α/β, PKA C, and STAT1, reducing their phosphorylation (Fig. 2E; S3A). Silencing PP2A-C restored PKA C, STAT1, and GSK3α/β phosphorylation, though not Akt, suggesting PP2A-independent regulation of Akt (Fig. S3B).

ANP32A deletion increased PP2A targeting of NFκB p65 and LYRIC (Fig. 2C; Fig. S3B), with corresponding declines in phosphorylation, reversible by PP2A-C silencing (Fig. 2F; S3E). NFκB p65 promoter binding decreased in ANP32A-deficient cells (Fig. 2H), confirming reduced transcriptional activity.

CIP2A knockout increased PP2A binding to STAT1/3, decreasing phosphorylation levels (Fig. 2G; S3C), with rescue upon PP2A-C inhibition (Fig. S3F). c-Myc levels also decreased following CIP2A deletion (Fig. 2I), consistent with reports implicating CIP2A in c-Myc stabilization [14]. Suggesting that in GBMs, CIP2A is a critical regulator of c-Myc in addition to EGFR and mTORC2 hyperactivation [22,23].

We next assessed phosphorylation of kinases and transcription factors in tumors derived from control and EIP-deficient cell lines. Loss of EIPs consistently resulted in reduced protein phosphorylation *in vivo*, supporting a critical role for these inhibitors in sustaining oncogenic signaling networks (Fig. S4). Together, these findings establish that EIPs suppress PP2A activity to maintain phosphorylation of oncogenic effectors.

### 3.3. EIPs promote radioresistance and facilitate radiation-induced activation of ATR and/or ATM in GBMs

**Fig. 3.**
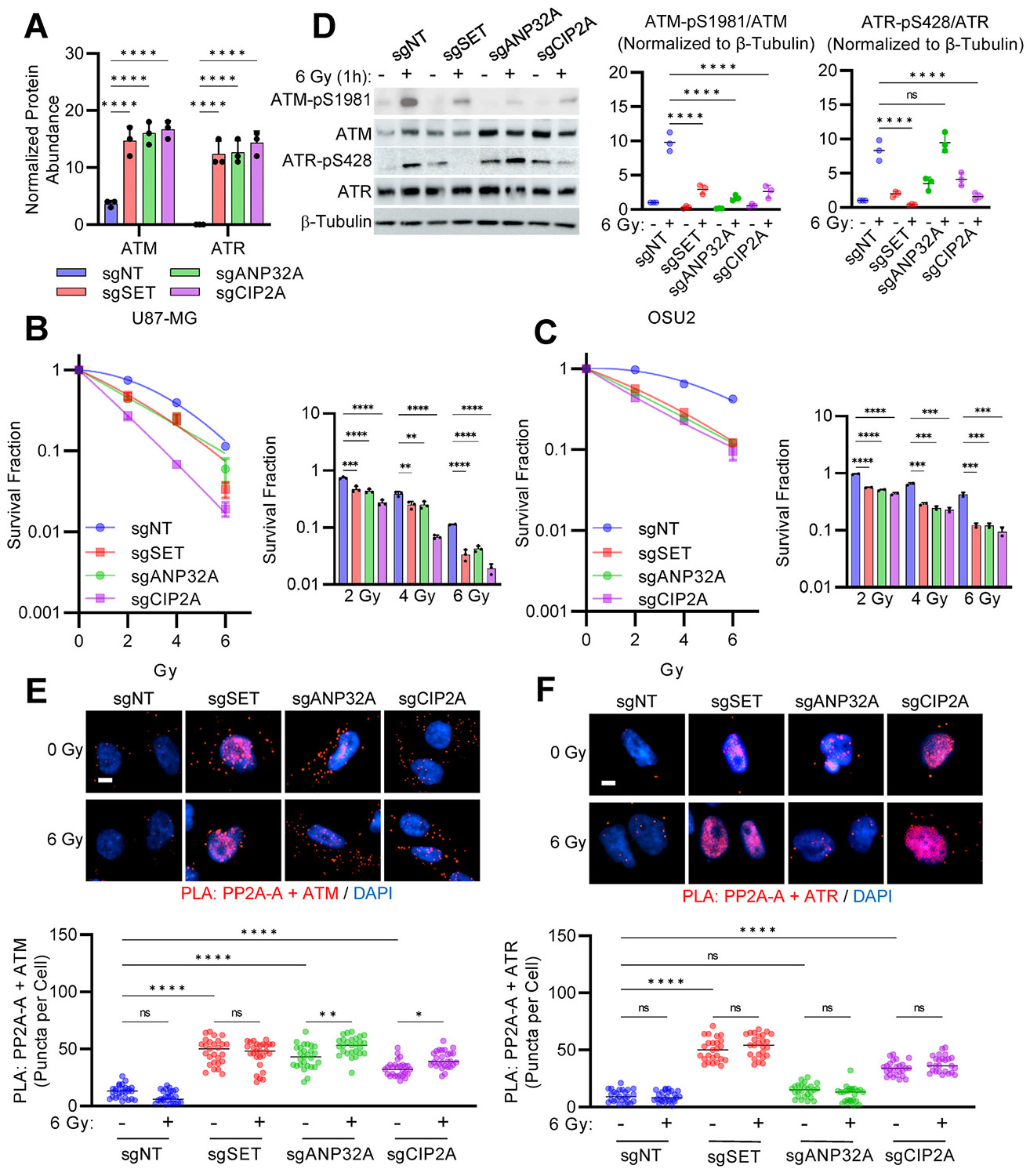
EIPs promote radioresistance and facilitate radiation-induced activation of ATR and/or ATM in GBMs. (a) Normalized ATM and ATR abundance from proteomic analysis of the PP2A interactome in the indicated cell lines. (b) Left: Clonogenic analysis of U87-MG sgNT, sgSET, sgANP32A, and sgCIP2A cell following radiation treatment at the indicated doses (n = 3). Right: Statistical analysis comparing the radiation sensitivity of U87-MG sgNT, sgSET, sgANP32A, and sgCIP2A cell lines. (c) Left: Clonogenic analysis of OSU2 sgNT, sgSET, sgANP32A, and sgCIP2A cells following radiation treatment at the indicated doses (n = 3). Right: Statistical analysis comparing the radiation sensitivity of OSU2 sgNT, sgSET, sgANP32A, and sgCIP2A cell lines. (d) Left: Assessment of indicated protein levels using Western blot from U87-MG sgNT, sgSET, sgANP32A, and sgCIP2A cells at basal levels and 1 h following 6 Gy radiation. Right: Quantification of ATM and ATR phosphorylation levels normalized to total protein levels and β-Tubulin (n = 3). (e) Top: Representative images of *in situ* co-localization of PP2A-A and ATM using proximity ligation assay in U87-MG sgNT, sgSET, sgANP32A, and sgCIP2A cells at basal levels and 1 h post 6 Gy radiation. Bottom: Quantification of puncta per cell (n = 50). Scale bar: 5 μm. (f) Top: Representative images of *in situ* co-localization of PP2A-A and ATR using proximity ligation assay in U87-MG sgNT, sgSET, sgANP32A, and sgCIP2A cells at basal levels and 1 h post 6 Gy radiation. Bottom: Quantification of puncta per cell (n = 50). Scale bar: 5 μm. Data represents mean ± SD. *p < 0.05, **p < 0.01, ***p < 0.001. See also Fig. S5.

Proteomic data showed increased PP2A association with ATM and ATR in EIP knockouts (Fig. 3A), suggesting a role in DNA damage response (DDR). Clonogenic assays confirmed that all EIP-deficient cells were more radiosensitive (Fig. 3B and C; S5A, B). SET and CIP2A deletion reduced radiation-induced ATR activation, and all three knockouts attenuated ATM phosphorylation (Fig. 3D). The reduction in ATM and ATR activation was associated with increased PP2A–ATM and PP2A-–ATR proximity integrated over time, as measured using our Bio-ID2–PP2A-Aα system (Fig. S5C and D). In contrast, proximity ligation assays, which detect acute and spatially restricted interactions, confirmed increased PP2A–ATM association following radiation, with SET loss preferentially enhancing nuclear interactions, whereas ANP32A and CIP2A loss favored cytoplasmic association (Fig. 3E and F).

Radiation did not disrupt these PP2A-ATM/ATR associations in knockouts. Though PP2A-DNA-PKcs interactions also increased in SET and ANP32A knockouts, phosphorylation levels remained unchanged post-radiation (Fig. S5F-G), suggesting ATM/ATR are the likely principal PP2A targets mediating radiosensitivity.

### 3.4. EIPs maintain DNA damage repair and G2/M checkpoint control following radiation

ATM and ATR activation in response to radiation is critical for initiating DNA repair and enforcing cell cycle checkpoints. To examine the functional consequences of impaired DDR kinase activation in EIP-deficient cells, we assessed single-strand (ssDNA) and double-strand (dsDNA) break repair following irradiation.

**Fig. 4.**
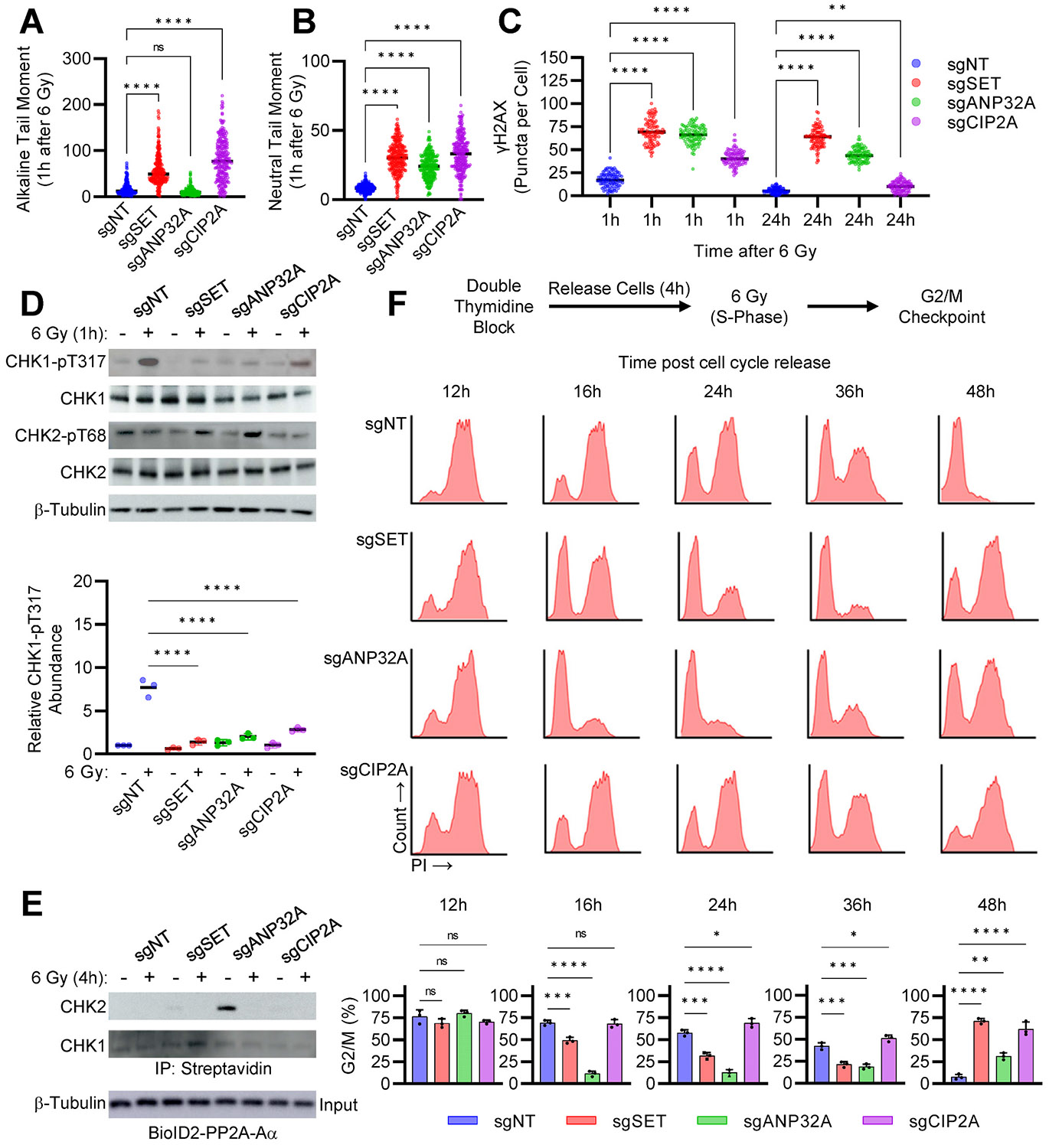
EIPs maintain DNA damage repair and G2/M checkpoint control following radiation. (a) Tail length of single stranded DNA breaks was measured in U87-MG sgNT, sgSET, sgANP32A, and sgCIP2A cells using the alkaline COMET assay 1 h following 6 Gy (n = 250 per condition). (b) Tail length of double stranded DNA breaks was measured in U87-MG sgNT, sgSET, sgANP32A, and sgCIP2A cells using the neutral COMET assay 1 h following 6 Gy (n = 250 per condition). (c) γH2AX puncta formation in U87-MG sgNT, sgSET, sgANP32A, and sgCIP2A cells 1 h and 24 h following 6 Gy (n = 250 per condition). (d) Top: Assessment of indicated protein levels using Western blot from U87-MG sgNT, sgSET, sgANP32A, and sgCIP2A cell lines at basal levels and in response to 6 Gy radiation 1 h following treatment. Bottom: Quantification of CHK1 phosphorylation levels normalized to CHK1 and β-Tubulin levels. (e) Assessment of indicated protein levels using Western blot of biotinylated proteins immunoprecipitated from U87-MG sgNT, sgSET, sgANP32A, and sgCIP2A cells transfected with BioID2-PP2A-Aα for 24 h followed by 6 Gy radiation. Lysates were taken 4 h post radiation. (f) Top: Cell cycle analysis of U87-MG sgNT, sgSET, sgANP32A, and sgCIP2A cells using propidium iodide labeling. Cells were synchronized to G1/early S using a double thymidine block, released for 4 h, and treated with 6 Gy radiation. Cell cycle phase was analyzed at the indicated timepoints post cell cycle release. Bottom: Quantification of cells in G2/M phase at the indicated timepoints. Data represents mean ± SD. *p < 0.05, **p < 0.01, ***p < 0.001. See also Fig. S6.

Alkaline COMET assays showed that SET and CIP2A knockout cells failed to repair ssDNA damage, unlike ANP32A-deficient or control cells (Fig. 4A; S6A). Neutral COMET assays revealed impaired dsDNA repair across all knockouts (Fig. 4B; S6B). Similarly, increased γH2AX foci were observed 1 h post-radiation confirmed unrepaired damage, which persisted at 24 h in SET and ANP32A knockouts (Fig. 4C; S6C).

We next examined whether EIP loss affects radiation-induced cell cycle checkpoints, given that ATR and ATM activate CHK1 and CHK2 to mediate cell cycle arrest in response to DNA damage. EIP loss impaired CHK1—but not CHK2—activation following radiation (Fig. 4D). This was not explained by changes in CHK1/CHK2 biotinylation by BioID2-PP2A-Aα (Fig. 4E), implicating upstream ATM/ATR suppression.

These findings suggest that enhanced PP2A targeting of ATR and/or ATM in EIP-deficient cells suppresses CHK1 activation following radiation. As CHK1 activity is essential for initiating the G2/M checkpoint in response to DNA damage, we next assessed whether CHK1 suppression in EIP knockout cells impaired G2/M checkpoint engagement. Synchronized cell cycle analysis showed that control cells entered prolonged G2/M arrest post-radiation, whereas SET and ANP32A knockouts bypassed this checkpoint and re-entered G1 within 24 h (Fig. 4F; S6D). CIP2A-deficient cells failed to proliferate, accumulated in G2/M, and exhibited mitotic abnormalities including multinucleation (Fig. S7A-B), consistent with checkpoint failure and mitotic slippage.

### 3.5. EIPs protect GBMs from radiation induced mitotic cell death and promote radioresistance independent of p53

**Fig. 5.**
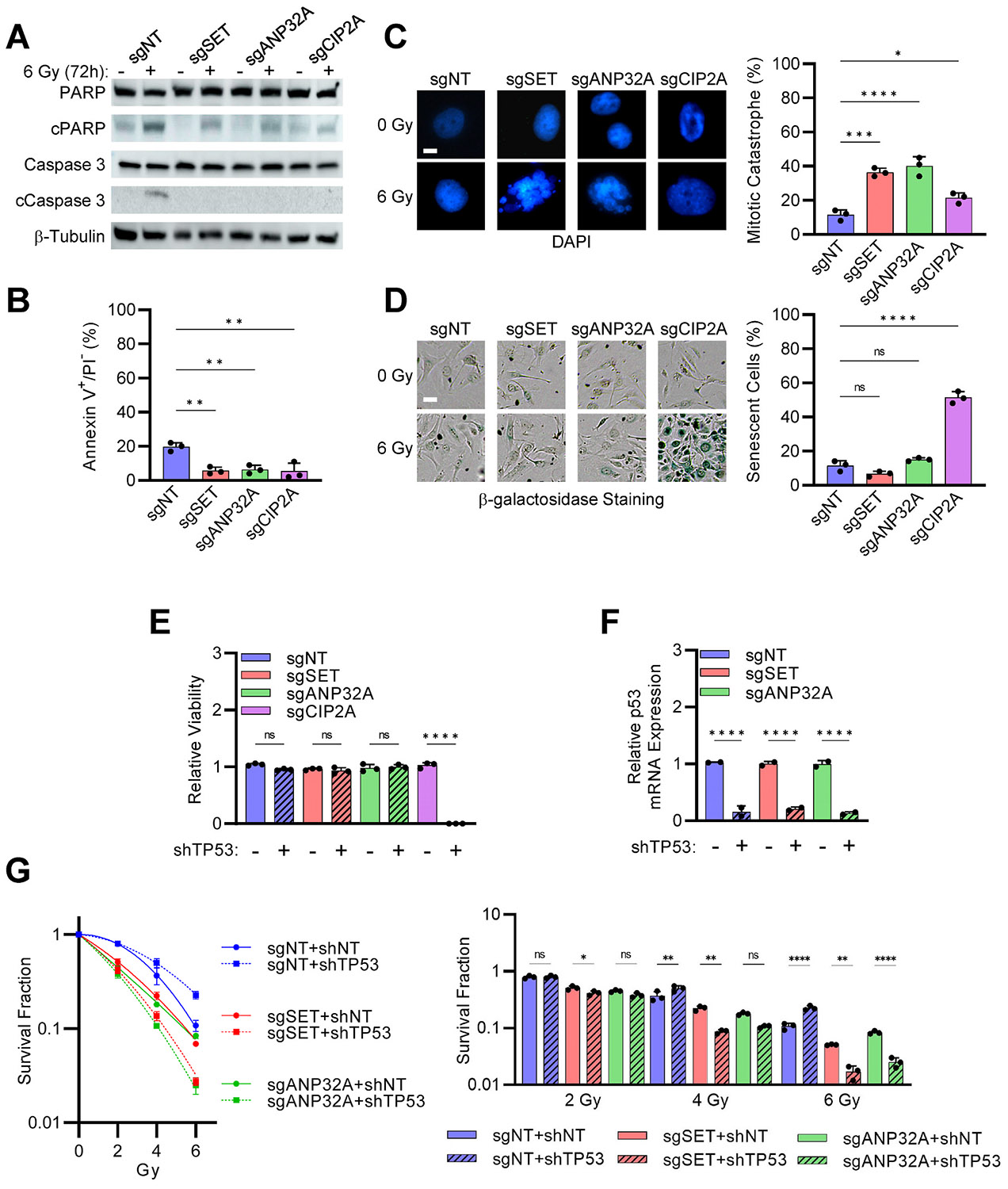
EIPs protect GBMs from radiation induced mitotic cell death and promote radioresistance independent of p53. (a) Assessment of indicated protein levels using Western blot of U87-MG sgNT, sgSET, sgANP32A, and sgCIP2A cells at basal levels and 72 h post 6 Gy radiation. (b) Quantification of apoptotic cells using annexin V + PI flow cytometry analysis of U87-MG sgNT, sgSET, sgANP32A, and sgCIP2A cells 72 h following 6 Gy radiation (n = 3). (c) Left: Representative images of U87-MG sgNT, sgSET, sgANP32A, and sgCIP2A cells 72 h post 6 Gy radiation. Scale bar: 5 μm. Right: Quantification of the percentage of cells with nuclear alterations (3^+^ lobes, 3^+^ microsatellites, 3^+^ nuclear fragmentations) consistent with mitotic catastrophe in U87-MG sgNT, sgSET, sgANP32A, and sgCIP2A cells (n = 100). (d) Left: Representative images of β-Galactosidase stained U87-MG sgNT, sgSET, sgANP32A, and sgCIP2A cells 7 days post 6 Gy radiation. Scale bar: 200 μm. Right: Quantification of the percentage of β-Galactosidase-positive cells (n = 100). (e) Relative viability of U87-MG sgNT, sgSET, sgANP32A, and sgCIP2A cells two weeks post transduction with lentivirus containing shRNA non-target (shNT) and shTP53 (n = 3). (f) mRNA levels of TP53 using RT-qPCR in U87-MG sgNT, sgSET, and sgANP32A cells transduced with shNT and shTP53 lentivirus (n = 3). (g) Left: Clonogenic analysis of U87-MG sgNT, sgSET, and sgANP32A cells transduced with shNT or shTP53 following radiation treatment at the indicated doses. Right: Statistical analysis comparing the radiation sensitivity of U87-MG sgNT, sgSET, and sgANP32A cells transduced with shNT or shTP53 cells. Data represents mean ± SD. *p < 0.05, **p < 0.01, ***p < 0.001. See also Fig. S7.

To elucidate the mechanisms of radiation-induced cell death in EIP-deficient cells, we examined both apoptotic and mitotic cell death pathways. Modest increases in cleaved PARP, with no corresponding activation of caspase-3, suggested limited apoptotic cell death following 6 Gy irradiation (Fig. 5A). Flow cytometric analysis using Annexin V/PI staining corroborated these findings, confirming minimal apoptosis (Fig. 5B). Instead, mitotic cell death increased in SET and ANP32A knockouts (Fig. 5C). Whereas CIP2A knockout induced multinucleation and senescence, confirmed by β-galactosidase staining (Fig. 5D; S7A, B).

To further examine this mechanism, we investigated the role of p53—a key regulator of radiation-induced cell cycle arrest and death—in modulating the radiosensitizing effects of EIP loss [24]. Given that ATM inhibitor-induced radiosensitization is influenced by p53 status in GBMs [25], we hypothesized that p53 deficiency might enhance radiosensitivity. To test p53 dependency, we silenced p53 in all cell lines. Only CIP2A-deficient cells showed selective cell death upon p53 loss (Fig. 5E and F), supporting a known synthetic lethality between p53 and CIP2A [26]. Clonogenic assays showed that p53 knockdown increased radioresistance in controls, but sensitized EIP-deficient cells—especially SET knockouts—to radiation (Fig. 5G). These findings align with previous work showing that p53 loss can reduce apoptosis-driven radiosensitivity in GBM [27], and with studies linking p53-deficient GBMs to increased sensitivity to ATM inhibition [25]. Overall, p53 status differentially regulates radiation responses in EIP-intact versus EIP-deficient GBM cells.

### 3.6. Inhibition of ATM or CHK1 selectively enhances the radiosensitization of control cells but not EIP knockout cells

**Fig. 6.**
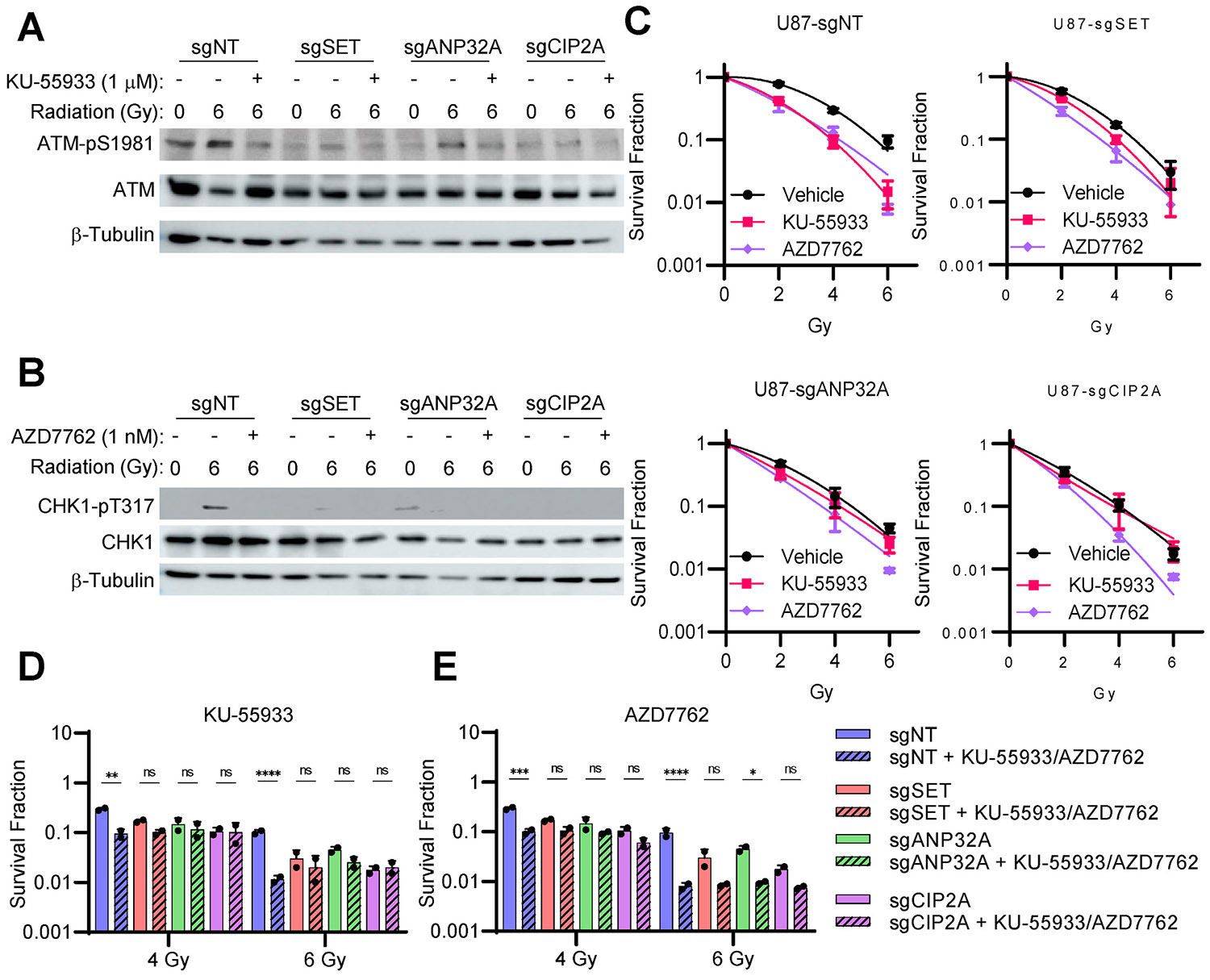
Inhibition of ATM or CHK1 selectively enhances the radiosensitization of control cells but not EIP knockout cells. (a) Assessment of indicated protein levels using Western blot of U87-MG sgNT, sgSET, sgANP32A, and sgCIP2A cells 1 h following 6 Gy radiation. 2 h prior to radiation, cells were pre-treated with vehicle (DMSO) or 1 μM KU-55933 (ATM inhibitor). (b) Assessment of indicated protein levels using Western blot of U87-MG sgNT, sgSET, sgANP32A, and sgCIP2A cells 1 h following 6 Gy radiation. 2 h prior to radiation, cells were pre-treated with vehicle (DMSO) or 1 nM AZD7762 (CHK1 inhibitor). (c) Clonogenic analysis of U87-MG sgNT, sgSET, sgANP32A, and sgCIP2A cells 2 weeks following radiation at the indicated doses. 2 h prior to radiation, cells were treated with vehicle (DMSO), or 1 μM KU-55933 or 1 nM AZD7762 (n = 3). (d) Statistical analysis comparing the radiation sensitivity of U87-MG sgNT, sgSET, sgANP32A, and sgCIP2A cells pre-treated with KU-55933 or vehicle (DMSO). (e) Statistical analysis comparing the radiation sensitivity of U87-MG sgNT, sgSET, sgANP32A, and sgCIP2A cells pre-treated with AZD7762 or vehicle (DMSO). Data represents mean ± SD. *p < 0.05, **p < 0.01, ***p < 0.001.

To confirm the contribution of ATM and CHK1 suppression to the heightened radiosensitivity in EIP-deficient cells, we evaluated the effects of pharmacological ATM and CHK1 inhibition in control and EIP knockout isogenic cell lines. We selected inhibitors and doses that did not affect baseline cell viability in the absence of radiation (Fig. 6A and B). In control cells, ATM inhibition significantly increased sensitivity to 4 and 6 Gy radiation. However, this radiosensitizing effect was absent in EIP knockout cells (Fig. 6C and D). CHK1 inhibition similarly enhanced radiosensitivity in control cells but had minimal impact on EIP-deficient lines, with the exception of ANP32A knockouts, which showed increased sensitivity at 6 Gy (Fig. 6C-E). These results indicate that EIPs contribute to radioresistance by preventing PP2A-mediated suppression of the ATM–CHK1 axis, thereby supporting efficient DNA repair and G2/M checkpoint control following radiation.

### 3.7. Combined inhibition of EIPs increases tumor suppressor efficacy and enhances radiation sensitivity in GBM

**Fig. 7.**
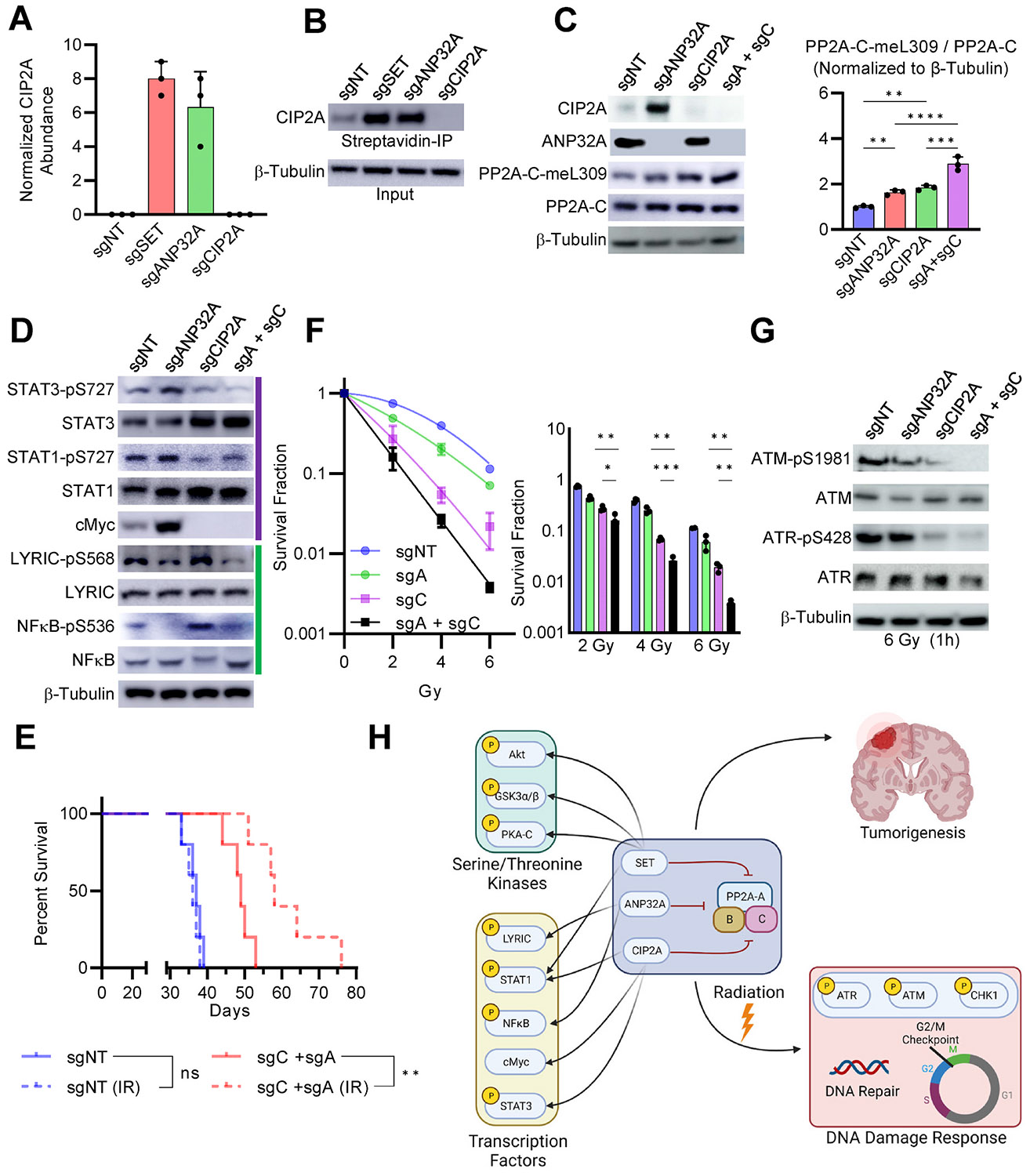
Combined inhibition of EIPs increase tumor suppressor efficacy and enhances radiation sensitivity in GBM. (a) Normalized CIP2A abundance from proteomic analysis of the PP2A interactome in the indicated cell lines. (b) Assessment of CIP2A protein levels using Western blot of biotinylated proteins immunoprecipitated from U87-MG sgNT, sgSET, sgANP32A, and sgCIP2A cells transfected with BioID2-PP2A-Aα for 24 h. (c) Left: Assessment of indicated protein levels using western blotting of U87-MG sgNT, sgANP32A, sgCIP2A, and sgANP32A + sgCIP2A cell lines. Right: Quantification of PP2A-C methylation levels normalized to total PP2A-C and β-Tubulin (n = 3). (d) Assessment of indicated protein levels using western blotting in the indicated cell lines. (e) Kaplan-Meier curve of mouse survival following intracranial implantation of U87-MG sgNT and sgANP32A + sgCIP2A cells (n = 5). Ten days post implantations, mice received 3 doses of 2 Gy radiation every other day for one week. (f) Left: Clonogenic analysis of the indicated cell lines following radiation treatment at the indicated doses (n = 3). Right: Statistical analysis comparing the radiation sensitivity of the indicated cell lines. (g) Assessment of indicated protein levels using western blotting 1 h after 6 Gy radiation in the indicated cell lines. (h) Graphic summary highlighting the oncogenic properties of SET, ANP32A, and CIP2A in GBM. sgA + sgC: sgANP32A + sgCIP2A double knockout cells. Data represents mean ± SD. *p < 0.05, **p < 0.01, ***p < 0.001. See also Fig. S7.

Lastly, our findings suggest a regulatory feedback loop among EIPs. Proteomic analysis revealed increased interaction between PP2A and CIP2A in SET and ANP32A knockout cells (Fig. 7A), which was validated by Western blot (Fig. 7B). To assess whether dual targeting of EIPs enhances tumor-suppressive activity, we generated double knockout cell lines. SET and CIP2A double knockout was not viable (Fig. 7C), whereas ANP32A and CIP2A double knockout resulted in an additive increase in PP2A activity. In ANP32A-deficient cells, elevated CIP2A–PP2A interaction correlated with increased activation of STAT1, STAT3, and total c-Myc, as well as NFκB and LYRIC. Combined loss of ANP32A and CIP2A suppressed activation of all five transcription factors (Fig. 7D).

In intracranial xenograft models, double knockout cells extended survival by 31%, compared to 13% and 20% for ANP32A and CIP2A knockouts, respectively (Fig. 1G; Fig. 7E). The combined knockout also enhanced radiosensitivity, as evidenced by reduced radiation-induced ATM and ATR activation (Fig. 7F and G). This radiosensitization was confirmed *in vivo*: mice bearing tumors derived from double knockout cells responded to fractionated radiation (3 × 2 Gy), while those with parental tumors showed no survival benefit (Fig. 7E). Together, these results demonstrate that combined targeting of EIPs may enhance the suppression of oncogenic signaling and augment tumor suppression as well as radiation response (Fig. 7H).

## 4. Discussion

Here, we demonstrate that GBMs suppress PP2A tumor suppressor activity through aberrant expression of the endogenous inhibitors ANP32A, CIP2A, and SET. This observation is consistent with prior studies implicating these factors in GBM pathogenesis [28-30]. Despite this prior work, the downstream phospho-signaling programs governed by individual EIPs—and their functional relevance to therapeutic response—have remained incompletely defined. Our study substantially extends this framework by identifying distinct, EIP-specific phospho-protein networks and by demonstrating that these signaling programs directly regulate radiation response. Collectively, these findings position endogenous PP2A inhibition not merely as a permissive oncogenic feature, but as a central determinant of signaling modularity and DNA damage tolerance in GBM.

At the signaling level, our data reveal that SET-dependent inhibition of PP2A is required to sustain activation of Akt, STAT1, and PKA-C, while concurrently suppressing GSK3α/β activity. While SET has previously been linked to oncogenic signaling in GBM [29], we are the first to demonstrate that SET-mediated PP2A inhibition maintains phosphorylation of GSK3α/β, PKA-C, and STAT1 in this context. The dependence of Akt activation on SET is supported by previous studies [31,32]. The marked reduction in tumorigenicity observed in U87-MG-sgSET cells is therefore likely attributable to the simultaneous disruption of several independent oncogenic signaling nodes—including Akt, PKA-C, and STAT1—each of which possesses tumor-promoting functions on its own [33-35]. These findings support a model in which SET functions as a signaling insulator, protecting multiple kinase pathways from PP2A-mediated dephosphorylation.

In parallel, we establish ANP32A as a critical regulator of NFκB and LYRIC signaling in GBM. The oncogenic role of ANP32A in GBM has been previously reported [28]; however, its downstream PP2A-regulated signaling targets had not been mechanistically defined. NFκB p65 is a well-established oncogenic transcription factor that promotes tumor growth, aggressiveness, and radiation resistance [36-38]. We further show that ANP32A prevents PP2A-mediated targeting of LYRIC, a transcription factor and RNA-binding protein previously demonstrated to promote astrocyte transformation and GBM tumorigenesis in xenograft models [39]. Increased interaction between PP2A and LYRIC following ANP32A loss resulted in decreased phosphorylation at serine 568. Notably, loss of serine 568 phosphorylation phenocopies LYRIC silencing [40], indicating that this phosphorylation site is critical for LYRIC activity. These findings provide mechanistic insight into how ANP32A-dependent PP2A inhibition sustains multiple oncogenic transcriptional programs in GBM.

We further demonstrate that CIP2A prevents PP2A-dependent inactivation of STAT1 and STAT3. This result is consistent with recent work showing that CIP2A and STAT3 form a positive feedback loop in GBM [41], thereby confirming and extending prior observations. In addition, we observed a marked reduction in cMyc protein levels following CIP2A knockout. Although cMyc was not identified in our unbiased proteomics screen, its functional relationship with CIP2A is well established [14]. These data support a model in which CIP2A sustains cMyc-driven transcription in GBM, acting in parallel with other reported regulatory mechanisms such as EGFR/EGFRvIII-mediated activation of mTORC2 and alternative splicing of the cMyc binding partner Max [22,23]. Together, these findings reinforce the concept that distinct EIPs stabilize non-redundant oncogenic signaling modules downstream of PP2A inhibition.

Beyond oncogenic signaling, our findings uncover a central role for EIPs in promoting radiation resistance in GBM. While CIP2A has previously been implicated in radioresistance through a CHK1–pSTAT3 feedback loop [41], we are the first to demonstrate that both SET and ANP32A also actively promote radioresistance. Mechanistically, we show for the first time that EIPs prevent PP2A-mediated targeting of ATM and ATR, thereby suppressing activation of these DNA damage response (DDR) kinases following irradiation. Although ANP32A has been reported to positively regulate ATM transcription in response to oxidative stress in normal tissues [42], we did not observe a reduction in total ATM protein levels following ANP32A knockout. This discrepancy suggests that ANP32A may regulate ATM through context-dependent mechanisms, including post-translational control in GBM.

Consistent with defective DDR signaling, impaired DNA repair and G2/M checkpoint control resulted in mitotic cell death following radiation in ANP32A-, CIP2A-, and SET-deficient cells. SET and ANP32A knockout cells predominantly underwent necrosis-like mitotic cell death characterized by extensive nuclear fragmentation and absence of apoptotic markers. In contrast, CIP2A knockout cells exhibited radiation-induced senescence, consistent with multiple prior reports [26]. These divergent cell fate outcomes likely reflect the distinct and partially overlapping phospho-protein networks protected from PP2A by each EIP.

We further demonstrate that EIP loss enhances radiosensitivity by disrupting DNA damage repair and G2/M checkpoint activation. These findings appear, at first glance, to contrast with reports showing radiosensitization by pharmacologic PP2A inhibition using LB-100 [43]. However, this apparent contradiction can be reconciled by the substrate- and context-dependent functions of PP2A. Our data indicate that EIP deletion radiosensitizes GBM cells through PP2A-mediated inhibition of ATM and/or ATR, leading to increased DNA damage burden and impaired checkpoint induction. In contrast, PP2A inhibition by LB-100 has been shown to enhance Plk1 and CDK1 phosphorylation, promoting inappropriate G2/M transition following radiation [44,45]. Thus, PP2A activation and inhibition may converge on radiosensitization by targeting distinct nodes within the DDR.

These findings highlight the therapeutic potential of targeting EIPs—particularly CIP2A—as a GBM-specific strategy to modulate DDR signaling. While DDR kinases are attractive therapeutic targets, their essential roles in normal tissues raise substantial concerns regarding therapeutic index. Inactivating mutations in ATM and DNA-PKcs cause severe radiation-induced normal tissue toxicity [46,47], and ATR is essential for somatic cell viability [48]. In contrast, CIP2A expression is largely restricted to GBM. Our data show that CIP2A loss enhances radiation sensitivity by preventing radiation-induced activation of ATM, ATR, and CHK1, supporting a model in which EIP targeting enables tumor-selective radiosensitization.

Our results also prompt reconsideration of other PP2A regulatory mechanisms. PME-1, a PP2A methylesterase, is reported to have heightened expression and oncogenic activity in GBM [49-51]. However, we observed a decrease in PME-1 expression at both the mRNA and protein levels in our analyses. We do not believe this observation contradicts prior reports but rather reflects differences in reference comparisons. Studies reporting elevated PME-1 in GBM typically compare relative expression across tumor grades, whereas our analysis incorporates normal brain tissue as a reference. Nonetheless, these findings raise the possibility that PME-1 may exert pro-tumorigenic effects independent of global overexpression.

Finally, this study has several important limitations that contextualize the scope of our findings. *In vivo* evaluation of EIP-dependent radiosensitization was constrained by biological viability requirements: SET-deficient GBM cells failed to establish tumors, and combined EIP loss markedly impaired baseline viability, precluding stable clonal isolation and systematic *in vivo* testing of dual or pan-EIP perturbations. Consequently, *in vivo* studies were restricted to individual EIPs that supported robust tumor formation while retaining reproducible radiosensitization *in vitro*. In addition, patient-derived glioma stem cell OSU2GSC failed to establish tumors in radiation-compatible mouse strains, necessitating the use of the U87-MG model for proof-of-principle *in vivo* validation. All *in vivo* experiments were conducted in immunodeficient hosts, precluding assessment of adaptive immune contributions, which is particularly relevant given emerging evidence that PP2A modulation can influence tumor immunogenicity and therapeutic response [52]. Therefore, future studies using immunocompetent or humanized GBM models will be required. Finally, our proteomic analyses revealed expanded PP2A engagement with metabolic and chromatin-associated proteins following EIP depletion, highlighting additional layers of PP2A regulation that warrant future investigation in immunocompetent and clinically relevant GBM models.

## Supplementary Material

1

2

## Appendix A. Supplementary data

Supplementary data to this article can be found online at https://doi.org/10.1016/j.canlet.2026.218325.
