# Supplementary material for: Endogenous inhibitors of PP2A activate oncogenic and DNA damage response kinases in glioblastoma
Source: Cancer Lett. Author manuscript; Available in PMC 2026 Jun 10. (PMC13250921; doi:10.1016/j.canlet.2026.218325)
Supplement: 1 [file NIHMS2178253-supplement-1.pdf]

Supplemental Information

Endogenous inhibitors of PP2A activates oncogenic and DNA damage response kinases in Glioblastoma.

John Ryan Jacob[1], Shahid M. Nimjee[2], J. Bradley Elder[3], Arnab Chakravarti[1#] and Kamalakannan Palanichamy[1#*]

[1]Department of Radiation Oncology, The Ohio State University College of Medicine and Comprehensive Cancer Center, Columbus, OH, 43210.

[2]Department of Neurosurgery, The Ohio State University Wexner Medical Center, Columbus, OH, 43210.

[3]Department of Neurological Surgery, The Ohio State University Wexner Medical Center, Columbus, OH, 43210.

[#]Senior Authors

*Correspondence & Lead Contact:

Kamalakannan Palanichamy, Goss Lab, Rm#359, 1925 Coffey Road, Columbus, OH 43210, USA.

Phone: 614-685-4245; Fax: 614-292-5435

Email: Kamalakannan.Palanichamy@osumc.edu

This document includes:

Supplemental Materials and Methods

Supplemental Figures S1 to S7

Supplemental Materials and Methods

Compounds and Antibodies

Primary antibodies Akt-pS473 (4060), Akt-pT308 (9275), Akt (4691), Erk1/2-pT202/Y204 (4370), Erk1/2 (4695), STAT3-pS727 (9134), STAT3 (4904), PKA C-pT197 (5661), PKA C (4782), STAT1-pS727 (8826), STAT1 (9172), NFκB p65-pS536 (3033), NFκB p65 (8242), cMyc (5605), GSK3α/β-S9/S21 (8566), GSK3α/β (5676), Caspase 3 (9662), PARP (9542), DNA-PKcs-p2056 (68716), DNA-PKcs (12311), ATM-pS1981 (13050), ATR-pS428 (2853), Chk1-p317 (12302), Chk1 (2360), Chk2-pT68 (2197), Chk2 (2662), γH2A.X (9718), Histone 3 (4499), PME-1 (29135), CIP2A (14805), ANP32A (15491), PP2A-C (2259), PP2A-A (2041), PPP2R2A (5689), PPP2R5D (5687), and PP2A B Subunit (2290) were purchased from Cell Signaling Technologies (CST, Beverly, Massachusetts, USA). SET (ab92872), PP2A-C-meL309 (ab66597), PPP2R2C (ab27269), and PPP2R3C (MBS2523402) were purchased from abcam (Dallas, Texas, USA). LYRIC-pS568 (630-080), LYRIC (13860-1-AP), ATM (MA1-23152), β-Tubulin (MA5-16308), PPP2R2B (PA5-29262), PPP2R5A (12675-2-AP), PPP2R5B (PA5-57740), and PPP2R5C (39-3600) were purchased from Thermo Fisher Scientific (Waltham, MA, USA). PPP2R2D (PA5-30763) was obtained from Invitrogen (Waltham, Massachusetts, USA). ATR (sc-515173) and PPP2R5E (sc-515676) were obtained from Santa Cruz (Dallas, Texas, USA). Secondary antibodies anti-rabbit IgG, HRP-linked (7074) and anti-mouse IgG, HRP-linked (7075) were purchased from Cell Signaling Technologies. Secondary antibody Donkey anti-Goat IgG (H+L) (Alexa Fluor 488) (A-11055) was purchased from Thermo. KU-55933 (587871-26-9) and AZD7762 (860352-01-8) were purchased from Selleckchem (Houston, Texas, USA).

Western Blotting

Cells were lysed in RIPA buffer (Thermo: J63306.AP) containing protease and phosphatase inhibitors (Thermo: 78440). Lysates were thoroughly mixed for 30 minutes on a rotator at 4 ºC. Lysates were then sonicated (Branson 450 Digital Sonifier) 10 times for 1 second at a 10% amplitude and cleared

via centrifugation at 12000 x g and 4 °C for 20 minutes. Protein levels were determined and loaded onto 10% SDS-PAGE gels and transferred onto 0.2 μM PVDF membranes. Membranes were blocked with 5% BSA for 1 hour at room temperature. Membranes were probed with primary antibodies in 5% BSA overnight at 4 °C. Membranes were washed three times in TBST for 5 minutes and probed with the appropriate horseradish peroxidase-labeled secondary antibody for 1 hour at room temperature. Signals were detected using Immobilon Western Chemiluminescent HRP Substrate (WBKLS0500) from Millipore (Burlington, Massachusetts, USA). To determine PP2A-C methylation levels, we adapted our western blotting protocol to prevent PP2A-C demethylation. Immediately after cell lysis, 90% of the lysate was aliquoted and denatured using 3:1 RIPA buffer to 4x Lamelli buffer containing 5% β-ME and boiled for 5 minutes at 95 °C . The remaining 10% was processed using our normal protocol and used to quantify the samples. The denatured samples were then normalized prior to western blotting.

The following was performed for normalization and quantification of phospho-protein signals. Total protein levels were first normalized to β-tubulin run on the same blot when possible. Phospho-protein signals were then normalized to their corresponding total protein levels, and final values are reported as phospho/total ratios normalized to β-tubulin. For high–molecular weight proteins (e.g., ATM and ATR), extended gel run times required for adequate protein resolution caused β-tubulin and other housekeeping proteins to migrate off the gel. In these cases, β-tubulin was run on a separate gel using lysates prepared from the same samples and processed in parallel. Phospho-protein, total protein, and β-tubulin samples were derived from the same lysate preparation and handled concurrently to minimize technical variability across blots.

CRISPR-Cas9

Lentivirus was purchased from Sigma Aldrich (St. Louis, MO, USA). U87-MG and OSU2 were transduced with pLv5-Cas9-Neo lentivirus (Sigma: CAS9NEO) at a MOI of approximately 10. Stable Cas9-expressing cells were established using geneticin selection for 7 days. Cells were then transduced with U6-gRNA:PGK-puro-2A-tagBFP lentivirus (Sigma) containing sgRNA sequences targeting SET (Clone ID: HS5000010041), ANP32A (Clone ID: HS5000019357), or CIP2A (Clone ID: HS5000015423)

at a MOI of approximately 10. Control cells were transduced with CRISPR-Lenti Non-Targeting Control Transduction Particles (Sigma: CRISPR12V-1EA) at the same MOI. Stable Cas9- and sgRNA-expression cells were established using puromycin selection for 7 days. Single cells were sorted into a 96-well plate using BD FACS Aria II for clonal selection.

Short-hairpin RNA

pLKO.1-puro transduction particles (Sigma) containing shRNA control (SHC016H) and shRNA TP53 (TRCN0000003753) were added to the U87-MG isogenic cell lines at an MOI of approximately 10. Cells were culture for 14 days and decreased TP53 expression was confirmed using RT-qPCR.

siRNA

About 1×10^5 cells per well were seeded in a 6-well plate. 24 hours after seeding, cells were treated with 100 nM small interfering RNA (siRNAs) mixed with 10 μL MISSION siRNA Transfection Reagent (sigma: S1452). siRNAs (sigma: 4390824) were obtained from Sigma targeting PPP2CA #1 (s10957) and PPP2CA #2 (s10959).

Real-time quantitative PCR (RT-qPCR)

Total RNA was isolated using the RNeasy Kit (74004) from Qiagen (Venlo, The Netherlands), per the manufacturer's protocol. For reverse-transcriptase reactions, first-strand cDNA was synthesized using Superscript II reverse transcriptase (Thermo: 18064022) per the manufacturer's protocol. TaqMan probes (Thermo) were used to estimate the gene expression level of TP53 (Hs00153349_m1). GAPDH (Hs99999 905_m1) and RNA18S1 (Hs03928990_g1) were used as housekeeping genes.

NFκB ELISA

About 2×10^6 cells were plated in a petri dish and incubated for 24 hours. The nuclear fraction was isolated using the Nuclear Extraction Kit (abcam: ab113474). The isolated nuclear faction was used to measure the transcriptional activity of NFκB with the NFκB p65 Transcription Factor Assay Kit (abcam: ab133112) according to the manufacturer's instructions. 25 μL of the provided Transcription Factor NFκB Competitor dsDNA was used to demonstrate specificity.

Clonogenics

Cells were plated at 2,000, 1,000, 500, and 50 cells/well in petri dishes and radiated at 6, 4, 2, and 0 Gy, respectively. The plates were incubated for 14 days. After 14 days, surviving colonies were stained with methylene blue, dried, and counted. Colonies of 50 cells were considered significant. The plating efficiency was calculated from the ratio of colonies formed over the number of cells plated.

COMET Assay

Cells were trypsinized and suspended in cold PBS at a concentration of $1x10^4$ cells/mL. The cells were then mixed with low melting agarose (Trevigen: 4250-050-02) at a ratio of 1:10 (v/v) and immediately plated onto Cometslides (Trevigen: 4252-040-01). For the alkaline comet assay, slides were immersed in Alkaline Unwinding solution (200 mM NaOH, 1 mM EDTA) for 1 hour at 4 °C. Electrophoresis was run at 21 V for 40 minutes at 4 °C in 1 mM EDTA pH>13 using the CometAssay Electrophoresis System (Trevigen: 4250-050-ES). Slides were washed 2x in water, dipped in 70% ethanol and dried in a 37 °C incubator. For the neutral comet assay, slides were incubated in neutral electrophoresis buffer (100 mM Tris Base, 300 mM Sodium Acetate, pH 9) for 30 minutes at room temperature. Electrophoresis was run at 21 V for 50 minutes at 4 °C in neutral electrophoresis buffer. Slides were then immersed in DNA precipitation buffer (1 M ammonium acetate, 70% Et-OH) for 30 minutes followed by incubation in 70% Et-OH for 30 minutes at room temperature. Slides were then dried in a 37 °C incubator. For both alkaline and neutral comet assays, DNA comets were detected using SYBR Gold Staining Solution (Thermo: S11494). Data were collected using a Cytation 5 imager. Comet tail moments were quantified using the OpenComet ImageJ plugin(1).

Annexin V / PI

To determine the number of apoptotic and necrotic cells, Annexin V/PI assays were performed using an apoptosis detection kit (Thermo: V13245). Briefly, cells were plated onto 6-well plates at a density of $2x10^5$ cells, incubated overnight, and irradiated. After incubation for the desired amount of time, the cells were harvested and washed in cold PBS. For every $1x10^5$ cells, 5 μL of Annexin V-FITC and 1 μL of 100 mg/mL PI were added to samples. Samples were incubated for 15 minutes at room temperature

in the dark and analyzed using flow cytometry. Analysis was conducted using FlowJo software.

RNA-Sequencing analysis of GBM (TCGA) and normal brain (GTEx) tissues

RNA-Sequencing data from TCGA and GTEx were integrated using the protocol outlined by Chen and MacDonald (2). GBM (TCGA) and normal brain tissue (GTEx) gene expression data was downloaded using the UCSCXenaTools package (3). Pathological data was used to remove IDH1/2 mutant samples from the GBM dataset. TCGA and GTEx data was combined using uniformly realigning reads to the hg38 genome, utilizing up-to-date protein-encoding gene list (4), and recalling RSEM values (5). Differential gene expression analysis was carried out using the voom method in the limma package (i.e., linear modelling and empirical Bayes moderation to assess differential expression and perform gene set testing) (6). The limma-voom methods assumes all samples have a similar range and distribution of log-CPM (counts per million reads) values. To meet this assumption, six samples from GTEx with a significantly different range and distribution were excluded. Data were subsequently normalized using the upperquartile method from the edgeR package (7).

Proximity Biotin Ligase

The BioID2-PP2A-Aα fusion protein was created using Gibson Assembly cloning kit (E5510S) from New England Biolabs (NEB, Ipswich, Massachusetts, USA). The PPP2R1A open reading frame, corresponding to protein PP2A-Aα, was obtained from Origene (SC121908 ; Rockville, MD, USA). The myc-BioID2-13x Linker-MCS plasmid (92308) was obtained from Addgene (Watertown, MA, USA). The myc-BioID2-13x Linker-MCS plasmid was digested using AscI restriction enzyme (NEB: R0558S). Subsequently, PPP2R1A ORF was amplified with primers containing overhangs to the complementary sequences in the myc-BioID2-13x Linker-MCS plasmid. The PPP2R1A ORF PCR product was isolated and purified from agarose gel using the QIAquick Gel Extraction Kit (Qiagen: 28704). The primer sequences used for Gibson cloning are listed below, capitalized letters indicate overhangs complimentary to myc-BioID2-13x Linker-MCS sequences.

PPP2R1A (Fwd): tagcggaggcggtggatcgggaATGGCGGCGGCCGACGGC

PPP2R1A (Rev): gctcgaggttcttgaaacggTCAGGCGAGAGACAGAACAGTCAGAGCCTCC

About $2x10^6$ U87-MG cells were transfected with 40 μg of the myc-BioID2-13x-Linker-PP2A-Aα plasmid using Lipofectamine 3000 (40 μL P3000, 40 μL Lipofectamine 3000). Cells were incubated for 24 hours followed by the addition of biotin (sigma: B4639) to a final concentration of 25 mM. Cells were incubated for an additional 24 hours to allow labeling. Cells were lysed and processed using the methods outlined in the western blotting methods. Biotinylated proteins were isolated using 600 μL Dynabeads MyOne Streptavidin C1 beads (Thermo: 65001) on a rotator overnight at 4 °C . Beads were collected and washed 2x with Wash buffer 1 (2% SDS in PBS) for 5 minutes on rotator. This was followed by three sequential washes with wash buffer 2 (pH 7.5, 0.1% deoxycholate, 1% Triton X-100, 500 mM NaCl, 1 mM EDTA), wash buffer 3 (pH 8.1, O.5% NP-40, 0.5% deoxycholate, 1 mM EDTA, 10 mM Tris) and wash buffer 4 (pH 7.4, 50 mM Tris, 50 mM NaCl) for 5 minutes at room temperature on a rotator. Biotinylated proteins immunoprecipitated by streptavidin were processed by the Proteomics Shared Resource at The Ohio State University Comprehensive Cancer Center for protein quantification and identification of protein interactors. For protein extraction for western blotting, beads were centrifuged at full speed for 1 minute at 4 °C. Protein was extracted for beads using 3:1 RIPA buffer to 4x Lamelli buffer containing 5% β-ME saturated with biotin. Samples were boiled at 95 °C for 5 minutes and cooled on ice for 5 minutes. Samples were separated from the magnetic beads and used for western blotting.

Mass Spectrometry–based Proteomics and Quantification

Streptavidin beads were washed three times with 50 mM ammonium bicarbonate and digested on-bead with sequencing-grade modified trypsin (Promega) at 37 °C overnight, with intermittent vortexing. Digestion was quenched by acidification with acetic acid, and peptides were collected for LC–MS/MS analysis.

Tandem mass spectrometry was performed using data-dependent acquisition (DDA) on a Thermo Fisher Scientific Orbitrap Fusion mass spectrometer equipped with a Nanospray FAIMS Pro source operated in positive ion mode. Tryptic peptides (4.0 µL) were separated on an Easy-Spray nanoLC

column (PepMap RSLC C18, 3 μm, 100 Å, 75 μm × 150 mm) using a 120-min gradient at a flow rate of 300 nL/min. Three FAIMS compensation voltages (−50, −65, and −80 V) were applied during data acquisition.

Raw data were searched using Mascot (version 2.7.0) via Proteome Discoverer (version 2.4) against the UniProt human database (20210604; 20,513 entries) and the cRAP contaminant database. Searches assumed strict trypsin specificity with up to four missed cleavages, a precursor mass tolerance of 10 ppm, and a fragment ion tolerance of 0.50 Da. Carbamidomethylation of cysteine was specified as a fixed modification, while oxidation of methionine and deamidation of asparagine and glutamine were specified as variable modifications.

Peptide and protein identifications were validated using Scaffold (version 4.11.0). Peptides were accepted at ≥96% probability to achieve a false discovery rate (FDR) <1%, and proteins were accepted at ≥99% probability with at least two unique peptides, based on the Protein Prophet algorithm.

Label-free protein quantification was performed using a spectral counting approach, in which relative protein abundance was estimated by comparing the number of MS/MS spectra assigned to each protein across samples. To account for differences in MS sampling depth between runs, total spectral count normalization was applied, whereby spectral counts for each protein were scaled to the total number of identified spectra per sample prior to quantitative comparison. The quantitative values reported therefore represent total spectral count–normalized spectral counts and were used for relative comparisons of protein abundance between conditions.

Because the bait protein (PPP2R1A) was expressed as a fusion protein for proximity labeling, its recovery and spectral counts may reflect a combination of expression level, proximity-dependent self-biotinylation, incorporation into PP2A complexes, and labeling efficiency, which may vary across biological conditions. Accordingly, bait spectral counts were not used as a normalization factor. Detection of the bait in streptavidin pulldowns is expected in proximity labeling experiments and confirms successful enrichment of bait-associated material.

Cell Cycle Synchronization and Analysis

About $1x10^5$ cells were seeded in a 6-well plate and incubate for 24 hours. Media containing 2 mM thymidine (Sigma: T1895) was added to cells for 20 hours. Cells were washed 4x with 2 mL PBS. Thymidine-free media was added for 9 hours. Cells were washed once with 2 mL PBS. Media containing 2 mM thymidine was added to cells for 20 hours. Cells were washed 4x with 2 mL PBS. Cells were then released by the addition of Thymidine-free media. Cells were trpsinized and processed for cell cycle analysis or western blot at the desired timepoints. For cell cycle analysis, cells were fixed in ice-cold 70% ethanol diluted in PBS. Cells were washed 2x with 1 mL ice-cold PBS followed by the addition 0.5 mL of FxCycle PI/RNase Staining Solution (Thermo: F10797). Cells were vortexed gently and incubated for 1 hour at room temperature in dark prior to flow cytometry analysis.

Immunofluorescence

About $1.5x10^4$ cells were plated per well in an 8-well chamber slide and incubated overnight. Cells were washed 2x with ice-cold PBS and fixed with 4% formaldehyde for 10 minutes. Cells were washed 2x with ice-cold PBS and permeabilized using 0.5% triton X-100 in PBS. Cells were incubated in blocking buffer (5% horse serum, 2% fish gelatin) for 60 minutes at 37 °C. Primary antibody was added in blocking buffer and incubated overnight at 4 °C on a rocker. Slides were washed 3x with PBST (0.1% Tween 20) and Invitrogen IgG (H+L) Highly Cross-Adsorbed Secondary Antibody was added at 1:1000 in blocking buffer. Cells were incubated for 1 hour at 37 °C and washed 1x with PBST. Slides were mounted with ProLong Diamond Antifade Mountant with DAPI (Invitrogen: P36971) and sealed. Imaging was conducted on Cytation 5 imager.

Proximity Ligation Assay

Cells were seeded, fixed, permeabilized and blocked as outlined in the immunofluorescence method. Proximity ligation assay was performed using the Duolink *in Situ* Detection Reagents Red (Sigma: DUO92008) according to manufacturer's instruction. Rabbit and mouse antibodies targeting our proteins of interest were added and incubated overnight at 4 °C on a rocker. Anti-mouse Plus probe (DUO92001) and anti-rabbit Minus probe (Sigma: DUO92005) were added to the samples for 1 hour at 37 °C . The ligase was added for 30 minutes followed by incubation with the polymerase for 100 minutes

at 37 °C . Amplification was quenched via multiple washes and slides were mounted and sealed. Images were acquired using the Cytation 5 imager (Biotek).

β-Galactosidase Assay

About $5x10^4$ cells were seeded in a six well plate and incubated for 24 hours. Cells were treated with 0 or 6 Gy radiation. Senescence was measured 7 days after radiation using the Senescence β-Galactosidase Staining Kit (CST: 9860) according to manufacturer's instruction. Briefly, cells were fixed and treated with 2.5 mM X-gal staining solution with pH 6. Plates were incubated overnight in a dry 37 °C incubator (no CO_2). Cells were imaged using phase microscopy on the Cytation 5 imager.

Immunohistochemistry

Paraffin-embedded section were deparaffinized via sequential 5-minute incubations in 100% Xylene, 100% Xylene, 100% Et-OH, 100% Et-OH, 95% Et-OH, 95% Et-OH, 50% Et-OH, and 100% water. Antigen retrieval and IHC steps were performed according to manufacturer's instructions (Dako, Glostrup, Denmark). Briefly, slides were placed in the PT link Pre-Treatment Module (Dako) with TRIS-EDTA (pH 9) buffer for 20 minutes at 97 °C. Slides were dipped in 1x Dako wash buffer (Dako: S3006) for 30 seconds and rinsed with water. Blocking solution (Dako: X0909) was added for 1 hour in a 37 °C chamber. Primary antibodies were added to Dako Antibody Diluent (S0809) and added to slides for 2 hours in a 37 °C chamber. Slides were incubated with Dako peroxidase block for 5 minutes and washed with water. Peroxidase labeled polymer specific to Rabbit (Dako: K4010) or Mouse (Dako: K4006) antibodies was added for 30 minutes at 37 °C. Slides were washed 2x with water. Dako substrate-chromogen was added for 10 minutes to develop slides. Slides were then washed with water and counterstained with Hematoxylin (Thermo: SH26-500D).

References

1. B. M. Gyori, G. Venkatachalam, P. S. Thiagarajan, D. Hsu, M. V. Clement, OpenComet: an automated tool for comet assay image analysis. *Redox Biol* **2**, 457-465 (2014).
2. H. M. Chen, J. A. MacDonald, Network analysis of TCGA and GTEx gene expression datasets

for identification of trait-associated biomarkers in human cancer. *STAR Protoc* **3**, 101168 (2022).
3. S. Wang *et al.*, UCSCXenaShiny: An R/CRAN Package for Interactive Analysis of UCSC Xena Data. *Bioinformatics* **38**, 527-529 (2021).
4. A. Piovesan *et al.*, Human protein-coding genes and gene feature statistics in 2019. *BMC Res Notes* **12**, 315 (2019).
5. J. Vivian *et al.*, Toil enables reproducible, open source, big biomedical data analyses. *Nat Biotechnol* **35**, 314-316 (2017).
6. C. W. Law *et al.*, RNA-seq analysis is easy as 1-2-3 with limma, Glimma and edgeR. *F1000Res* **5** (2016).
7. M. D. Robinson, D. J. McCarthy, G. K. Smyth, edgeR: a Bioconductor package for differential expression analysis of digital gene expression data. *Bioinformatics* **26**, 139-140 (2010).

Supplemental Figures S1 to S7

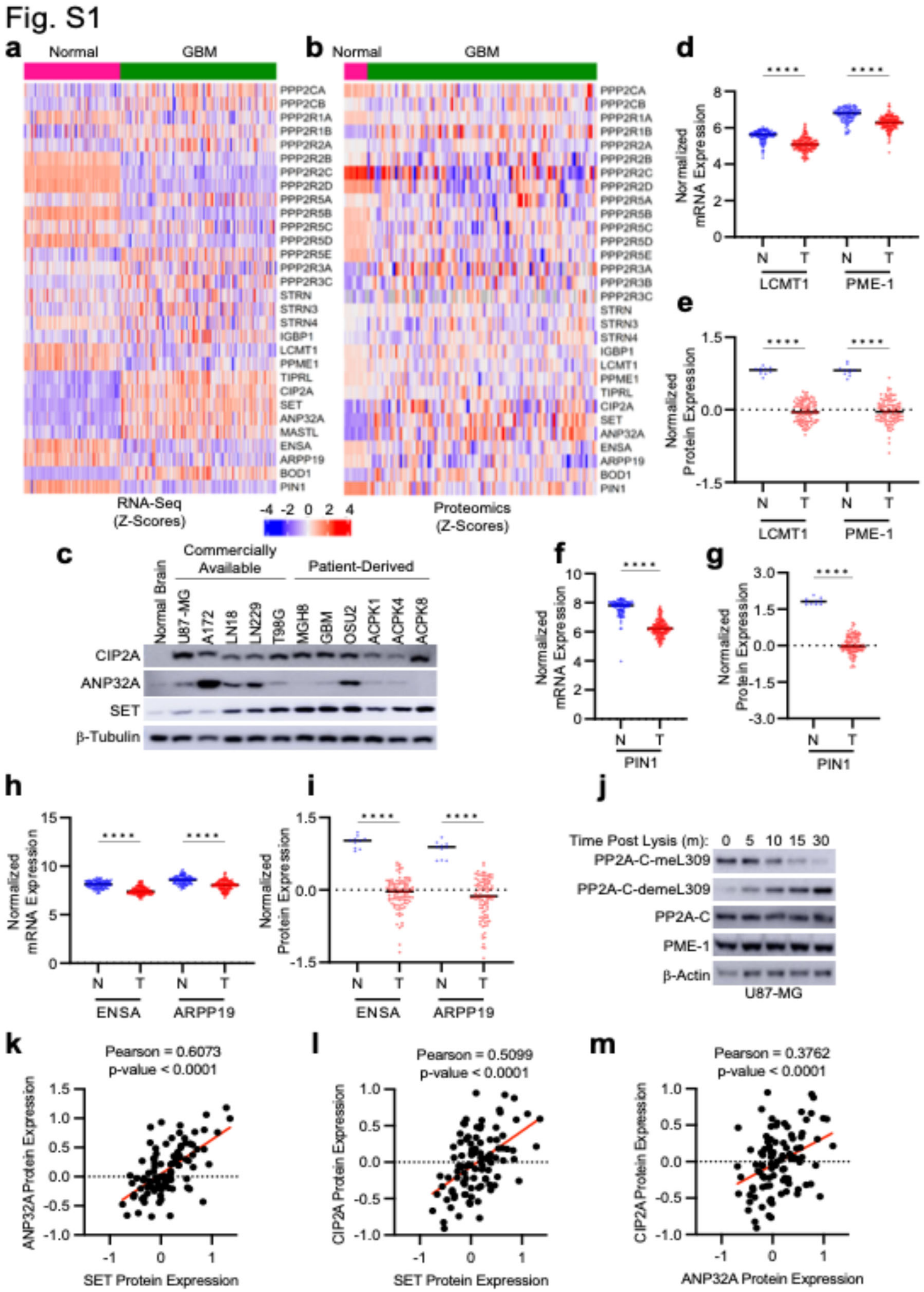

Supplemental Figure S1. Expression profiles of PP2A subunits and regulators in GBM and normal brain tissues.

(A) Heatmap representing the mRNA expression of PP2A subunits and regulators in normal brain (GTEX, n=105) compared to IDH1/2 wild-type GBM (TCGA, n=172) tissues. (B) Heatmap representing the protein levels of PP2A subunits and regulators in normal brain (n=10) compared to IDH1/2 wild-type GBM (n=99) tissues (Wang et al., Cancer Cell, 2021). (C) Assessment of indicated protein levels using western blotting across a panel of established and patient-derived GBM cell lines. (D, F, H) Relative mRNA expression of indicated genes from normal brain (GTEx, n=105) and GBM (TCGA, n=172) tissues. (E, G, I) Relative expression of indicated proteins from normal brain (n=10) and GBM (n=99) tissues (Wang et al., Cancer Cell, 2021). (J) Assessment of indicated protein levels using western blotting of U87-MG lysates denatured at the indicated timepoints following cell lysis. (k) Pearson correlation analysis comparing protein expression levels of SET and ANP32A across GBM samples. (l) Pearson correlation analysis comparing protein expression levels of SET and CIP2A across GBM samples. (m) Pearson correlation analysis comparing protein expression levels of ANP32A and CIP2A across GBM samples.

Fig. S2

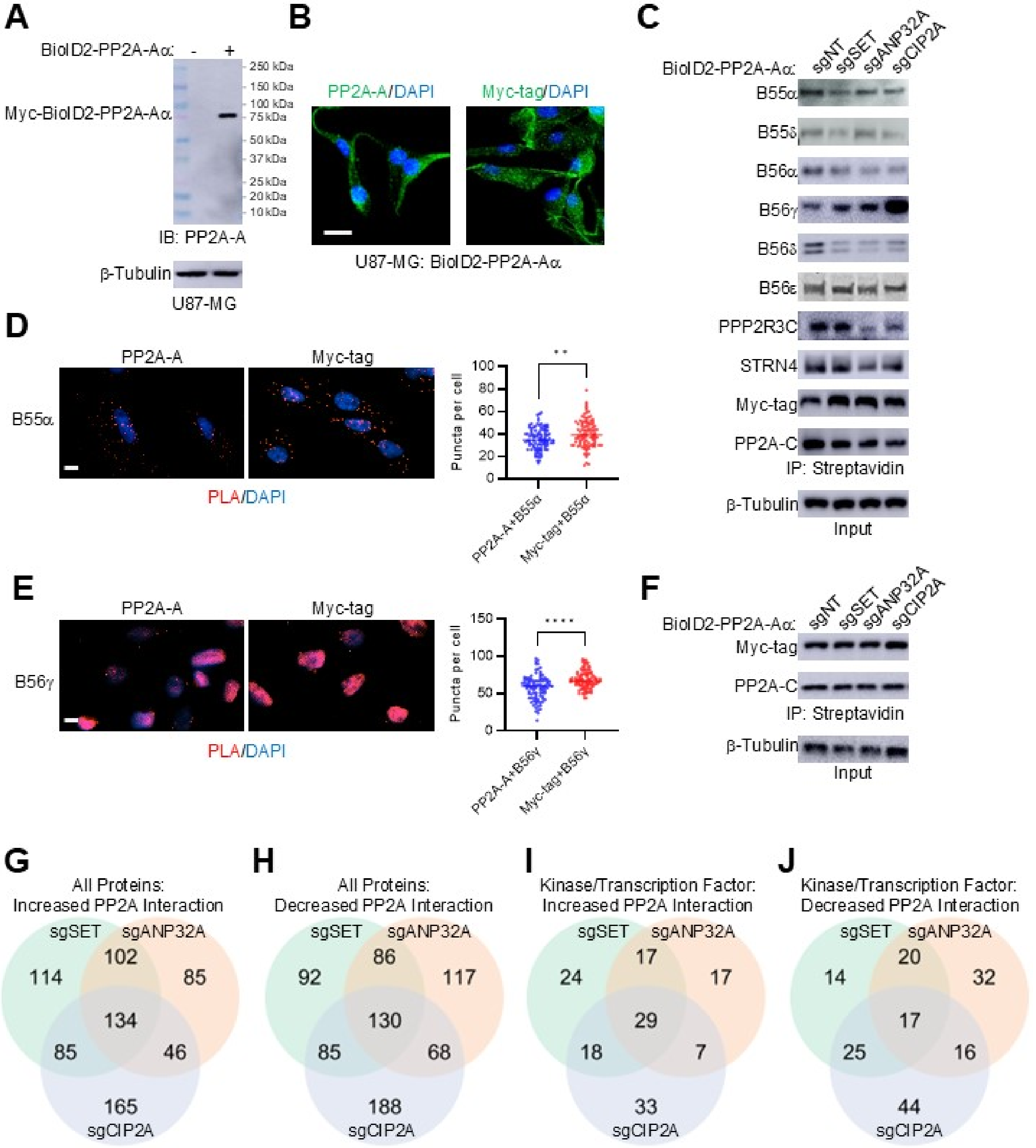

Supplemental Figure S2. Unbiased analysis of the PP2A interactome identifies oncogenic signaling nodes controlled by EIPs. (A) Assessment of BioID2-PP2A-Aα fusion protein stability using western blot 48 hours after transfection of U87-MG cells. (B)

Immunofluorescence analysis of the subcellular localization of the BioID2-PP2A-Aα fusion protein and endogenous PP2A-A 48 hours after transfection of U87-MG cells. Scale bar: 5 μm. (C) Assessment of indicated protein levels using western blot of proteins labeled via biotinylation by the BioID2-PP2A-Aα fusion protein and isolated using streptavidin beads. Protein levels were assessed in the U87-MG sgNT, sgSET, sgANP32A, and sgCIP2A cell lines. (D) Left: Representative images of *in situ* co-localization of B55α and BioID2-PP2A-Aα (myc-tag) or endogenous PP2A-A using proximity ligation assay in U87-MG cells. Right: Quantification of puncta formation (n=100 per condition). Scale bar: 5 μm. (E) Left: Representative images of *in situ* co-localization of B56γ and BioID2-PP2A-Aα (myc-tag) or endogenous PP2A-A using proximity ligation assay in U87-MG cells. Right: Quantification of puncta formation (n=100 per condition). Scale bar: 5 μm. (F) Assessment of indicated protein levels using western blot of proteins labeled via biotinylation by the BioID2-PP2A-Aα fusion protein and isolated using streptavidin beads in the indicated cell lines. Represented samples were submitted for proteomics analysis of the PP2A interactome. (G) Venn diagram of all proteins identified to have an increased interaction with PP2A in U87-MG sgNT, sgSET, sgANP32A, and sgCIP2A cell lines. (H) Venn diagram of all proteins identified to have a decreased interaction with PP2A in U87-MG sgNT, sgSET, sgANP32A, and sgCIP2A cell lines. (I) Venn diagram of kinases and transcription factors identified to have an increased interaction with PP2A in U87-MG sgNT, sgSET, sgANP32A, and sgCIP2A cell lines. (J) Venn diagram of kinases and transcription factors identified to have a decreased interaction with PP2A in U87-MG sgNT, sgSET, sgANP32A, and sgCIP2A cell lines. *P* values, Student's t test. Error bars, sd. *: $p<0.05$, **: $p<0.01$, ***: $p<0.001$.

Fig. S3

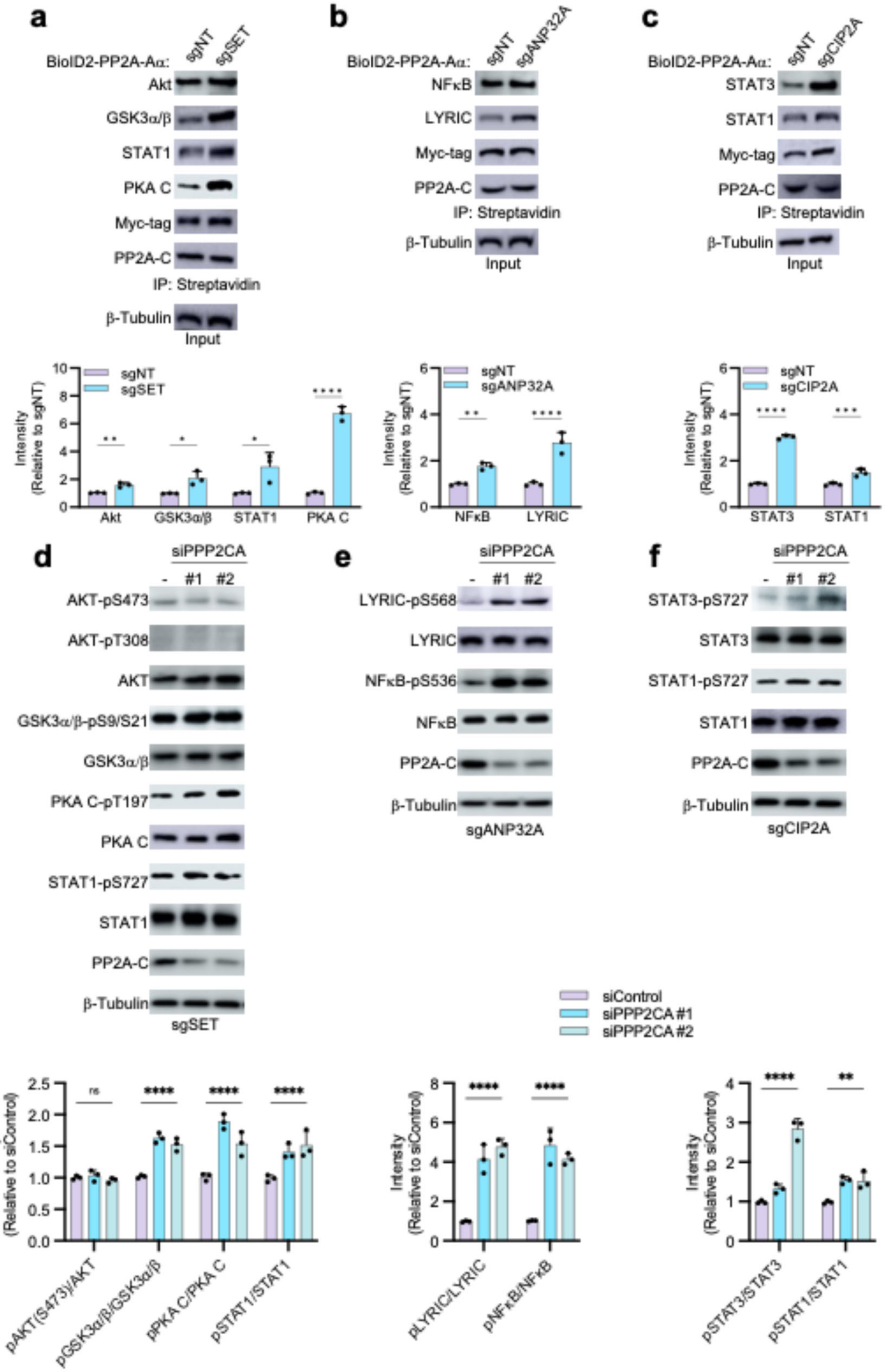

a
BioID2-PP2A-Aα:
sgNT
sgSET
Akt
GSK3α/β
STAT1
PKA C
Myc-tag
PP2A-C
IP: Streptavidin
β-Tubulin
Input
b
sgANP32A
NFκB
LYRIC
c
sgCIP2A
STAT3
d
siPPP2CA
AKT-pS473
AKT-pT308
AKT
GSK3α/β-pS9/S21
PKA C-pT197
STAT1-pS727
e
LYRIC-pS568
NFκB-pS536
f
STAT3-pS727
siControl
siPPP2CA #1
siPPP2CA #2
Intensity (Relative to sgNT)
Intensity (Relative to siControl)
pAKT(S473)/AKT
pGSK3α/β/GSK3α/β
pPKA C/PKA C
pSTAT1/STAT1
pLYRIC/LYRIC
pNFκB/NFκB
pSTAT3/STAT3

Supplemental Figure S3. EIP-induced changes in oncogenic phosphoprotein activation are rescued by PP2A inhibition. (A-C) Top: Assessment of indicated protein levels using western blot of proteins labeled via biotinylation by the BioID2-PP2A-Aα fusion protein and isolated using streptavidin beads. Protein levels were compared between U87-MG sgNT and (A) sgSET, (B) sgANP32A, and (C) CIP2A cell lines. Bottom: Quantification of the indicated proteins (n=3). (D-F) Top: Assessment of indicated protein levels using western blot of proteins isolated following 8 hours treatment with 100 nM siPPP2CA in isogenic U87-MG cell lines (D) sgSET, (E) sgANP32A, and (F) sgCIP2A. Bottom: Quantification of the indicated proteins (n=3). *P* values, Student's t test. Error bars, sd. *: $p<0.05$, **: $p<0.01$, ***: $p<0.001$.

Fig. S4

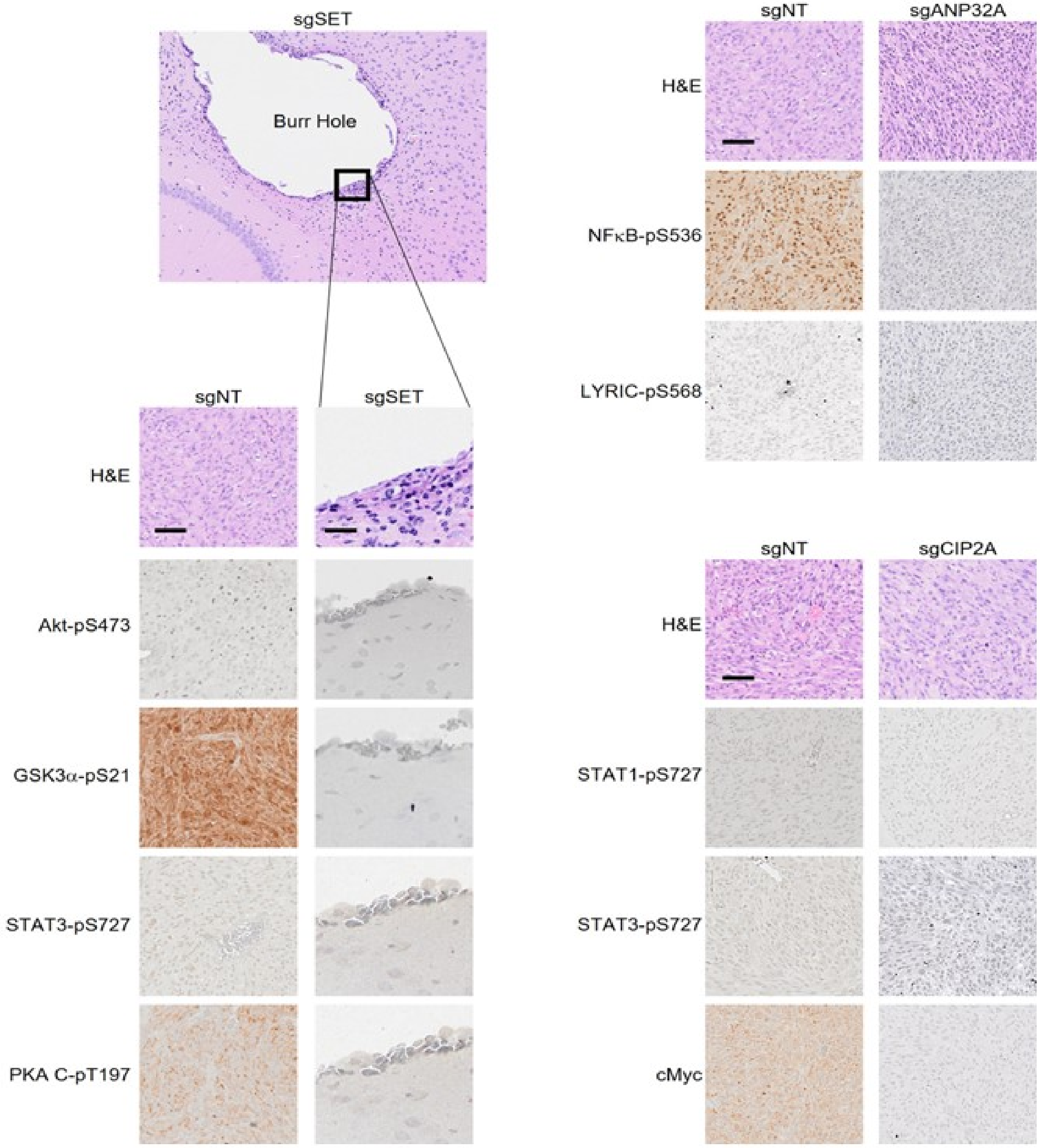

Supplemental Figure S4. Immunohistochemistry of kinase and transcription factor activation in tumors derived from EIP knockout cell lines. Representative hematoxylin and eosin (H&E) staining and immunohistochemical analysis of orthotopic tumors derived from non-targeting control (sgNT) or EIP-deficient (sgSET, sgANP32A, sgCIP2A) GBM cell lines. Left, low-magnification H&E image of an

sgSET implantation site showing the burr hole and tumor boundary, with higher-magnification insets highlighting tumor regions analyzed by immunohistochemistry. Tumor sections were stained with antibodies recognizing phosphorylated Akt (pS473), GSK3α (pS21), STAT3 (pS727), PKA C-subunit (pT197), NF-κB (pS536), LYRIC (pS568), STAT1 (pS727), and total c-Myc in the indicated EIP knockout and control tumors. Scale bars equal 50 μm.

Fig. S5

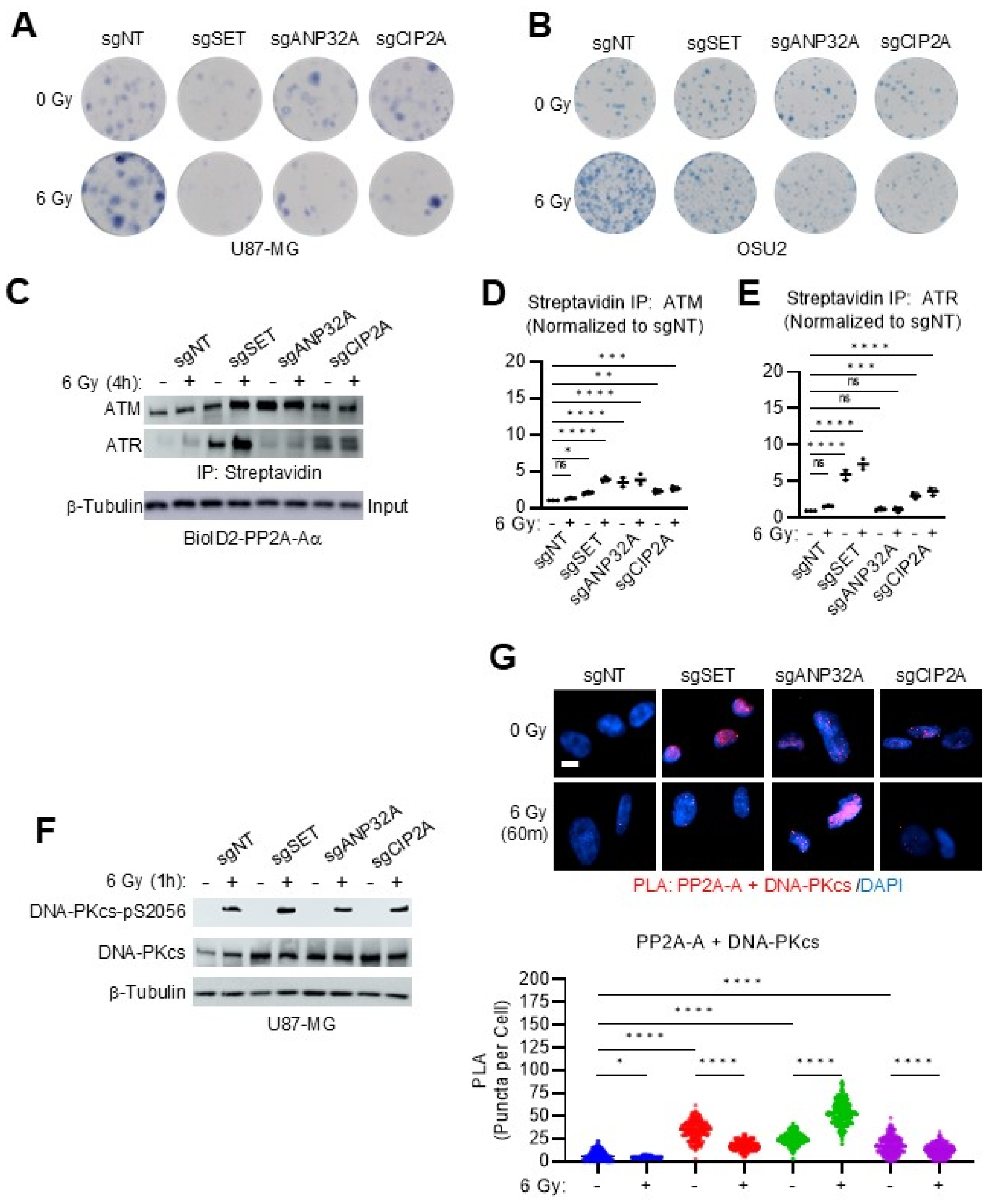

Supplemental Figure S5. EIPs promote repair of radiation-induced DNA damage and prevent ATM and ATR inactivation by PP2A. (A) Representative images of the colony forming ability of U87-MG sgNT, sgSET, sgANP32A, and sgCIP2A cells 2 weeks following 0 Gy or 6 Gy treatment. (B) Representative images of the colony forming ability of OSU2 sgNT, sgSET, sgANP32A, and sgCIP2A cells 2 weeks following

0 Gy or 6 Gy treatment. (C) Assessment of indicated protein levels using western blot of biotinylated proteins immunoprecipitated from U87-MG sgNT, sgSET, sgANP32A, and sgCIP2A cell lines transfected with BioID2-PP2A-Aα fusion protein for 24 hours followed by 6 Gy radiation. Lysates were taken 4 hours post radiation. (D) Quantification of total ATM immunoprecipitated from cells transfected with BioID2-PP2A-Aα for 24 hours followed by 6 Gy radiation. Lysates were taken 1 hour post radiation. ATM levels were normalized to non-irradiated control cells (sgNT-0Gy) (n=3). (E) Quantification of total ATR immunoprecipitated from cells transfected with BioID2-PP2A-Aα for 24 hours followed by 6 Gy radiation. Lysates were taken 1 hour post radiation. ATR levels were normalized to non-irradiated control cells (sgNT-0Gy) (n=3). (F) Assessment of indicated protein levels using western blot from U87-MG sgNT, sgSET, sgANP32A, and sgCIP2A cell lines at basal levels and in response to 6 Gy radiation 1 hour following treatment. (G) Top: Representative images of *in situ* co-localization of PP2A-A and DNA-PKcs using proximity ligation assay in U87-MG sgNT, sgSET, sgANP32A, and sgCIP2A cell lines at basal levels and 1 hour post 6 Gy radiation. Bottom: Quantification of puncta per cell (n = 250). Scale bar: 10 μm. *P* values, Student's t test. Error bars, sd. *: $p<0.05$, **: $p<0.01$, ***: $p<0.001$.

Fig. S6

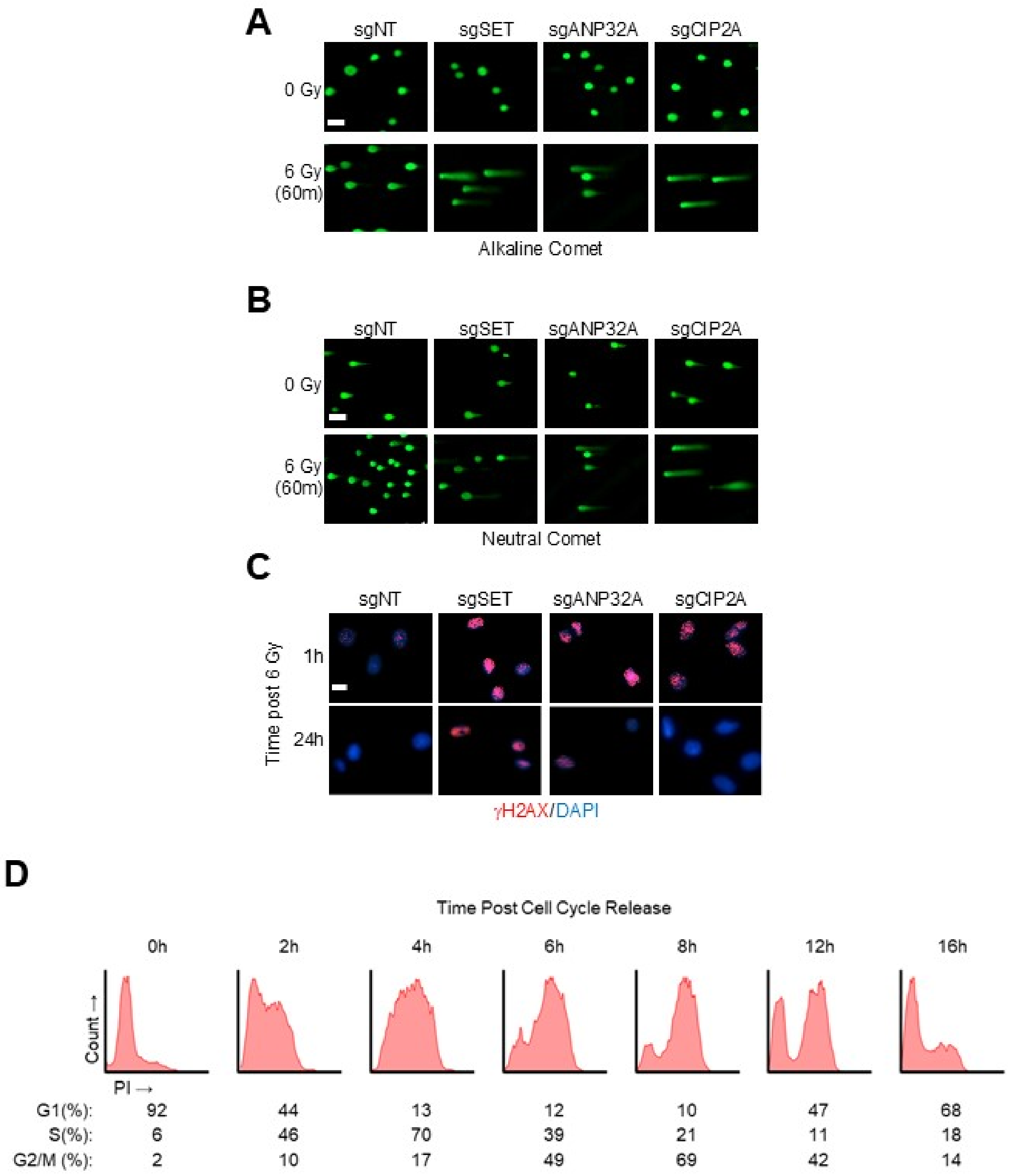

Supplemental Figure S6. EIPs facilitate DNA damage repair following radiation and cell cycle analysis optimization. (A) Representative images of alkaline COMET assay measuring single stranded DNA breaks in U87-MG sgNT, sgSET, sgANP32A, and sgCIP2A cells 1 hour following 6 Gy (n=250 per condition). Scale bar: 40 μm. (B) Representative images of neutral COMET assay measuring double stranded DNA

breaks in U87-MG sgNT, sgSET, sgANP32A, and sgCIP2A cells 1 hour following 6 Gy (n= 250 per condition). Scale bar: 40 μm. (C) Representative images of γH2AX immunofluorescence of U87-MG sgNT, sgSET, sgANP32A, and sgCIP2A cells 1 hour and 24 hours following 6 Gy (n= 250 per condition). Scale bar: 20 μm. (D) Cell cycle analysis using propidium iodine labeling of U87-MG cells following release from double thymidine block.

Fig. S7

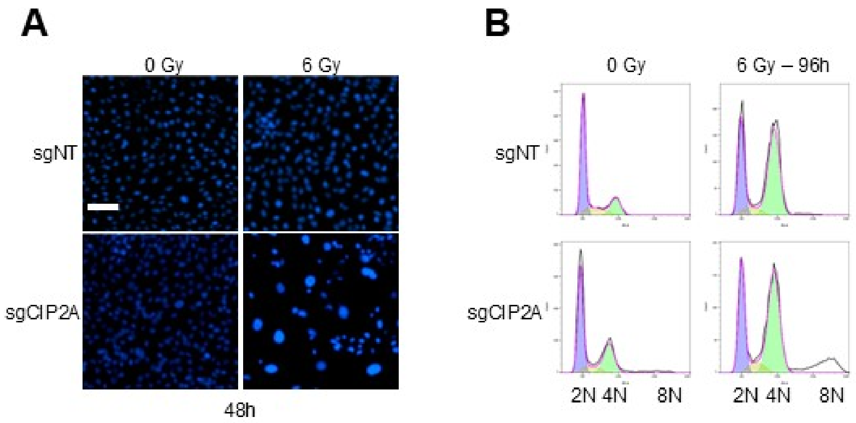

Supplemental Figure S7. Radiation induced multinucleation of CIP2A knockout cells. (A) Immunofluorescence images of DAPI nuclear staining of U87-MG sgNT and sgCIP2A cells 48 hours following 6 Gy. Scale bar: 200 μm. (B) Propidium iodine labeling of U87-MG sgNT and sgCIP2A cells 96 hours following 6 Gy.
